# Evaluation of residue-residue contact prediction methods: From retrospective to prospective

**DOI:** 10.1371/journal.pcbi.1009027

**Published:** 2021-05-24

**Authors:** Huiling Zhang, Zhendong Bei, Wenhui Xi, Min Hao, Zhen Ju, Konda Mani Saravanan, Haiping Zhang, Ning Guo, Yanjie Wei

**Affiliations:** 1 University of Chinese Academy of Sciences, Beijing, China; 2 Centre for High Performance Computing, Shenzhen Institutes of Advanced Technology, Chinese Academy of Sciences, Shenzhen, China; 3 Cloud Computing Department, Alibaba Group, Hangzhou, China; 4 College of Electronic and Information Engineering, Southwest University, Chongqing, China; Icahn School of Medicine at Mount Sinai, UNITED STATES

## Abstract

Sequence-based residue contact prediction plays a crucial role in protein structure reconstruction. In recent years, the combination of evolutionary coupling analysis (ECA) and deep learning (DL) techniques has made tremendous progress for residue contact prediction, thus a comprehensive assessment of current methods based on a large-scale benchmark data set is very needed. In this study, we evaluate 18 contact predictors on 610 non-redundant proteins and 32 CASP13 targets according to a wide range of perspectives. The results show that different methods have different application scenarios: (1) DL methods based on multi-categories of inputs and large training sets are the best choices for low-contact-density proteins such as the intrinsically disordered ones and proteins with shallow multi-sequence alignments (MSAs). (2) With at least 5L (L is sequence length) effective sequences in the MSA, all the methods show the best performance, and methods that rely only on MSA as input can reach comparable achievements as methods that adopt multi-source inputs. (3) For top L/5 and L/2 predictions, DL methods can predict more hydrophobic interactions while ECA methods predict more salt bridges and disulfide bonds. (4) ECA methods can detect more secondary structure interactions, while DL methods can accurately excavate more contact patterns and prune isolated false positives. In general, multi-input DL methods with large training sets dominate current approaches with the best overall performance. Despite the great success of current DL methods must be stated the fact that there is still much room left for further improvement: (1) With shallow MSAs, the performance will be greatly affected. (2) Current methods show lower precisions for inter-domain compared with intra-domain contact predictions, as well as very high imbalances in precisions between intra-domains. (3) Strong prediction similarities between DL methods indicating more feature types and diversified models need to be developed. (4) The runtime of most methods can be further optimized.

## Author summary

The amino acid sequence of a protein ultimately determines its tertiary structure, and the tertiary structure determines its function(s) and plays a key role in understanding biological processes and disease pathogenesis. Protein tertiary structure can be determined using experimental techniques such as cryo-electron microscopy, nuclear magnetic resonance and X-ray crystallography, which are very expensive and time-consuming. As an alternative, researchers are trying to use *in silico* methods to predict the 3D structures. Residue contact-assisted protein folding paves an avenue for sequence-based protein structure prediction and therefore has become one of the most challenging and promising problems in structural bioinformatics. Over the past years, contact prediction has undergone continuous evolution in techniques. Through a retrospective analysis of traditional machine learning /evolutionary coupling analysis methods/ consensus machine learning methods and a multi-perspective study on recently developed deep learning methods, we explore the most advanced contact predictors, pursue application scenarios for different methods, and seek prospective directions for further improvement. We anticipate that our study will serve as a practical and useful guide for the development of future approaches to contact prediction.

## Introduction

Residue-residue contacts refer to the residue pairs that are close within a specific distance threshold in the three-dimensional protein structure. Protein contact maps are “simplified” 2D representations of the 3D protein structure and are being considered as one of the most important components in modern protein structure prediction packages[1–15]. The application of predicted residue contacts has been extended to protein topology prediction[16], potential 3D model scoring and filtering[17,18], protein-protein interaction prediction[19–21]. Residue contacts can also be used as the distance restraints to accelerate the procedure of molecular dynamics simulations[22,23] and to predict the binding affinity in docking simulations[24].

Accurate residue contact prediction and its corresponding applications have been one of the most challenging and promising problems in structural bioinformatics. Early contact prediction methods are mainly based on mutual information(MI), mathematical optimization techniques, and traditional machine learning algorithms. Local statistical models such as MIp and MIc are unable to minimize the effect of transitive correlations since a residue pair is treated statistically independent of others. Physical constraints can also be used with integer linear programming techniques for residue contact prediction [25,26]. These ab initio contact prediction methods do not rely on evolutionary information, which can be a good solution for proteins lacking sequence homologs but can also significantly reduce the prediction accuracy for proteins with abundant homologs. Traditional machine learning methods like SVMcon[27], NNcon[28], SVMSEQ[29], SPINE-2D[30] predict the contact map matrix based on the pairwise strategy, ignoring the correlation among contacts, so these methods still show unsatisfactory prediction especially for long-range contacts. Methods combining integer linear programming with machine learning techniques[31,32] can also improve contact prediction performance in complementary ways, however, the improvement is not a significant leap compared to traditional machine learning methods.

Global statistical inference methods such as direct coupling analysis (DCA)[33] and sparse inverse covariance estimation (PSICOV) [34] achieve a breakthrough in capturing the correlated pattern of coevolved residues. These methods emphasize the importance of distinguishing between directly and indirectly correlated residues. A wide array of methods has been developed based on the ideas of DCA. EVfold (mfDCA) [35] is one variant of DCA implemented by mean-field approximations to inference with discrete variables. plmDCA[36], GREMLIN[37] and CCMpred[38] learn the direct couplings as parameters of a Probabilistic Graphical Model (Markov random field) by maximizing its pseudo-likelihood. gDCA[39], replacing the discrete amino acid variables with continuous Gaussian random variables, is a very efficient multivariate Gaussian modeling variant of DCA. Freecontact[40] is a fast replacement for EVfold (mfDCA) and PSICOV and also contains many fine-tunable parameters that can have different effects on the prediction results. DCA-based methods show higher accuracy compared to the MI methods or traditional machine learning methods when deep MSAs are available. While various studies have been conducted on how to disentangle indirect coupling among residues, COLORS[41] removes background correlations mainly caused by phylogenetic biases through low-rank and sparse decomposition (LRS) of a residue correlation matrix. These techniques based on evolutionary coupling analysis (ECA) assume that contacting residue pairs should present correlated mutations in the long-term evolutions reflected in the MSA, but frequently become powerless for targets with a limited number of homologous sequences. To further increase accuracy and recall, consensus-predictors like PconsC[42], MetaPSICOV[43], RRCRank and NeBcon[44] combine the output of different ECA-based or ML-based contact predictors to create consensus predictions.

Significant progress in accurate contact prediction has been achieved by integrating evolutionary coupling analysis (ECA) and deep neural networks. Deep learning (DL) based methods like DeepCov[45] and PconsC4[46] use pure MSA as input, which greatly reduces the complexity and calculation time of the prediction models. Whereas, their prediction accuracies will also be greatly affected by the number of effective sequences in the MSA. When effective sequences in the MSA are not adequate, DL methods that integrate different kinds of information as input features would become more successful. Methods in this category include RaptorX-Contact[47], DeepContact[48], DeepConPred2[49], DNCON2[50], DEEPCON [51], SPOT-Contact[52], DeepCDpred[53], ResPRE[54] and MapPred[55]. Since the introduction of large-scale language models for natural language processing, there has been considerable interest in developing similar models for proteins. AlphaFold2 at CASP14 applied an attention-based neural network system-Transformer to residue space. The original AlphaFold weighed all distances equally, in comparison, the attention-based network can identify which edges are important. Rives et al. [56] and Rao et al. [57]used transformer attention maps to perform unsupervised contact prediction, and the models show better performance than the best ECA methods such as GREMLIN or CCMpred. DL based contact prediction has recently demonstrated unprecedented ability to assist protein structure reconstruction in packages such as RaptorX[13], trRosetta[14] and AlhpaFold[15].

Though the assessment of residue contact predictors has been conducted in CASP for a long time [58,59], there are only a limited number of targets for the contact assessment session and the prediction result provided by different groups are not based on the same inputs. Wuyun et al.[60] and de Oliveira et al. [61] evaluated 15 ECA/ML methods and 8 ECA methods respectively on large sets of protein chains with the same inputs. In the rising wave of artificial intelligence applications, more and more deep-learning-based contact prediction methods have been developed. However, there is no comparative assessment on these DL methods with other categories of methods through large-scale benchmark data sets and the same inputs. To this end, it is necessary to conduct a comprehensive review and a critical assessment of current methods from different perspectives.

In this study, we assess 18 locally installed contact predictors (as the representatives of current methods) on three data sets (an independent test set of 610 proteins, a test set of 215 proteins and 32 CASP13 targets) according to a wide range of perspectives. As listed in S1 Table, the evaluated methods cover several different categories including traditional ML, consensus ML, ECA, single-input DL and multi-input DL methods. Compared to previous assessment works, the novelties of this study lie in: (1) Our assessment not only contains the common aspects (influence of sequence length & effective MSA, protein structural class, method similarity, etc.) as previous works, but also incorporates perspectives related to model probability, contact density, physical chemistry property, protein domains, distribution dispersion and running time. (2) Even for the common aspects, we also try to provide observations from more different angles. For example, the MSA search is performed against the largest protein sequence database (NCBI-nr) to better uncover the impact of effective MSA on prediction performance and applicable conditions of different methods; we analyze the performance of different methods on protein structural classes with special attention on intrinsically disordered and multi-domain proteins. (3) A comprehensive and critical evaluation on DL based methods that play vital roles in contact/tertiary structure prediction, which has not been previously conducted on large-scale datasets with the same inputs yet; the evaluation is not isolated, but treated as part of the evolution of contact prediction techniques by revisiting the traditional ML, ECA and consensus ML methods. (4) The predicted contacts from different methods are applied to tertiary structure reconstruction based on a large-scale test set consisting of highly non-redundant proteins from different structural classes. Through the large-scale evaluation, we aim to investigate factors that significantly affect the performance, explore the most advanced predictors, pursue application scenarios for different methods, and seek prospective directions for further improvement.

## Materials and methods

### Benchmark data set

We use three test sets for analysis: (a) 610 non-redundant protein chains from Protein Data Bank, indicated as TestSet1; (b) 215 non-redundant protein chains from TestSet1, indicated as TestSet2; and (c) 32 CASP13 domain targets, indicated as TestSet3. TestSet1 is obtained using the following steps: (1) 3136 protein chains are left through culling from PDB using PISCES[17,18] with the maximum sequence identity of 20%, the maximum R-factor of 0.3, the minimum sequence length of 50 and resolutions better than 2.0 Å; (2) 610 protein chains are selected through culling the 3136 chains against the training sets of SVMcon (424 proteins), NNcon (424 proteins), MetaPSICOV (624 proteins), COLORS (150 proteins), DeepCov(3456 proteins), PconsC4 (2891 proteins), DeepConPred2 (3443 proteins), DNCON2 (1230 proteins), SPOT (10200 proteins) with the cutoff of 20% sequence identity. TestSet2 containing 215 protein chains which are obtained through further culling TestSet1 against the training set of trRosetta (15051 proteins) and RaptorX (11410 proteins). TestSet2 is the subset of TestSet1. TestSet1 and TestSet2 are used for the evaluation of 16 methods (without trRosetta and RaptorX) and all 18 methods, respectively. TestSet3 are 32 single domain targets of CASP13 from the CASP Prediction Center (http://predictioncenter.org/download_area/CASP13/targets/casp13.targets.TD.4public.tar.gz).

### Definition of contact and contact density

In this study, the definition of residue-residue contact is directly taken from the CASP experiments. A pair of residues in the experimental structure is considered to be in contact if the distance between their Cβ atoms (Cα for Gly) is less than or equal to 8Å. Depending on the separation of two residues along the sequence (seq_sep), the contacts are classified into four classes: all-range (seq_sep≥6), short-range (6≤seq_sep<12), medium-range (12≤seq_sep<24) and long-range (seq_sep>24).

Contact density for each protein is measured by dividing the total number of non-local contacts (residue pair with seq_sep≥6) by the protein length[62,63].

### Number of effective sequences

Generating high-quality MSA is the first step for many contact prediction methods based on the fact that interacting residue pairs are under evolutionary pressure to maintain the structure. For the sake of establishing a fair comparison, all ECA and DL predictors use the same MSA as input for the same target protein. All results in this study use JackHMMER[64] searching against the nr database with iteration = 3 and E-value = 0.0001.

To better evaluate the impact of the number of effective sequences(*N*_*eff*_) on residue contact prediction, we calculate *N*_*eff*_ as depicted by Morcos et al.[35]. For an MSA = $\left\{(A_{1}^{a},A_{2}^{a}\ldots ,A_{L}^{a})|a=1,2\ldots ,M\right\}$ with sequence length L, the number of effective sequences is calculated as:

$$N_{eff}=\sum_{a=1}^{M}1/m^{a}$$ Eq 1

where M is the column number of MSA, *m^a^* is a number determined by $m^{a}=|b\in \{1,2\ldots M\}|{seq}_{identity(A^{a},A^{b})}>Threshold|$. In this work, the *Threshold* is defined as 68%.

### Criteria for evaluation

1. The predicted residue contact map is a matrix of probability estimates. We analyze the performance of predictors on reduced lists of contacts (sorted by the probability/score estimates) selected by either the probability/score threshold or the top L/*n* (L is the sequence length, and *n* = 1, 2, 5) criteria. The prediction performance is assessed using precision (accuracy in some references), coverage (recall in some references) and Matthew’s Correlation Coefficient (MCC), defined as follows:
$$Precision=\frac{N_{corr}}{N_{pred}}=\frac{TP}{TP+FP}$$ Eq 2
$$Coverage=\frac{N_{corr}}{N_{native}}=\frac{TP}{TP+FN}$$ Eq 3
$$MCC=\frac{TP\times TN-FP\times FN}{\sqrt{\left(TP+FP)(TP+FN)(TN+FP)(TN+FN\right)}}$$ Eq 4
  where *N*_*corr*_ is the number of correctly predicted contacts (physicochemical interactions or secondary structure interactions) in top L/*n* all-range (short-/ medium-/ long-range) contact predictions, *N*_*pred*_ is the number of predicted contacts (physicochemical interactions or secondary structure interactions) in top L/*n* all-range (short-/ medium-/ long-range) predicted contacts, and *N*_*native*_ is the number all-range (short-/ medium- /long-range) contacts (physicochemical interactions or secondary structure interactions) in the native structure. TP, FP, TN and FN are the number of true positive, false positive, true negative and false negative contacts (physicochemical interactions or secondary structure interactions) in top L/*n* all-range (short-/ medium- /long-range) predicted contacts, respectively.
2. Standard deviation reflects the degree of dispersion among individuals within the group, which is defined as:
$$STD=\sqrt{\frac{1}{N}\sum_{i=1}^{N}{\left(x_{i}-\bar{x}\right)}^{2}}$$ Eq 5
  where $\bar{x}$ is the mean of the variable *x*. The standard deviation can be used to evaluate the dispersion of *Precision*, *Coverage* and *MCC*.
3. Pearson correlation coefficient (*PCC*) is used to measure the strength and direction of the relationship between two variables. The correlation coefficient is obtained using the following formula:
$$PCC=\frac{\sum_{i=1}^{N}\left(x_{i}-\bar{x})(y_{i}-\bar{y}\right)}{\sqrt{\sum_{i=1}^{N}{\left(x_{i}-\bar{x}\right)}^{2}}\sqrt{\sum_{i=1}^{N}{\left(y_{i}-\bar{y}\right)}^{2}}}$$ Eq 6
  where $\bar{x}$ and $\bar{y}$ represent the sample means of the variables *x* and *y*. *PCC* = 1 means a perfect positive correlation and *PCC* = -1 means a perfect negative correlation.
4. Jaccard index (Jaccard similarity coefficient) measures the similarities between sets. It is defined as:
J(X,Y)=|X∩Y|/|X∪Y| Eq 7
  where *X* and *Y* are the sets of predicted contacts from two different predictors, |*X*∩*Y*| is the number of elements in the intersection of *X* and *Y*, and |*X*∪*Y*| represents the number of elements in the union of *X* and *Y*. The Jaccard index has values in the range of [0,1], with the value of 0 for completely dissimilar ones and 1 for identical predictors.
  The Jaccard index between two methods is calculated by averaging the Jaccard index value of each protein on the whole test set. The dendrogram heatmap in this study is calculated by applying Ward’s hierarchical clustering method to the Jaccard index matrix of all the methods.

## Results

In this section, we comprehensively assess the performance of 18 methods, namely SVMcon, NNcon, SVMSEQ, PSICOV, EVfold, FreeContact, plmDCA, gDCA, CCMpred, COLORS, MetaPSICOV, DeepCov, PconsC4, DeepConPred2, DNCON2, SPOT, trRosetta and RaptorX. The evaluation is conducted by considering a wide variety of factors such as contact density, model probability, structural types, domain complexity, prediction distribution, *N*_*eff*_ of the MSA, physicochemical properties, prediction similarity and runtime, etc. The results for Section 3.10 are based on the CASP13 protein targets. To be consistent with the CASP evaluation scheme, most sections (Section 3.1–3.2, Section 3.4 and Section 3.6–3.8) use the results of top L/*n* (*n* = 1,2,5) predictions. Section 3.3 considers the results given by specific probability/score thresholds, and the results in Section 3.5 & 3.10 combine both the top L/*n* and probability threshold schemes. The majority of evaluation results and analyses for Section 3.1–3.9 are based on TestSet1, and the results based on TestSet2 are mainly presented as supporting data in Section 3.1–3.2 and Section 3.6–3.8. This is due to the following reasons: (1) The data in TestSet1 is more abundant and diverse than that in TestSet2. (2) trRosetta and RaptorX show close overall prediction precisions (Section3.1) and high prediction similarities (Section 3.2) with SPOT. (3) The data in TestSet2 is not rich enough to meet the analysis needs of Section 3.3–3.5. (4) Similar conclusions could be drawn by comparing the results on TestSet1 with that on TestSet2 (Section 3.6, Section 3.7 and Section 3.8). Section 3.11 presents the results for contact-guided 3D structure reconstruction. The prediction results used in this study are available at http://hpcc.siat.ac.cn/hlzhang/RR-Contact/.

### The overall prediction precisions for the whole test set

**Fig 1:**
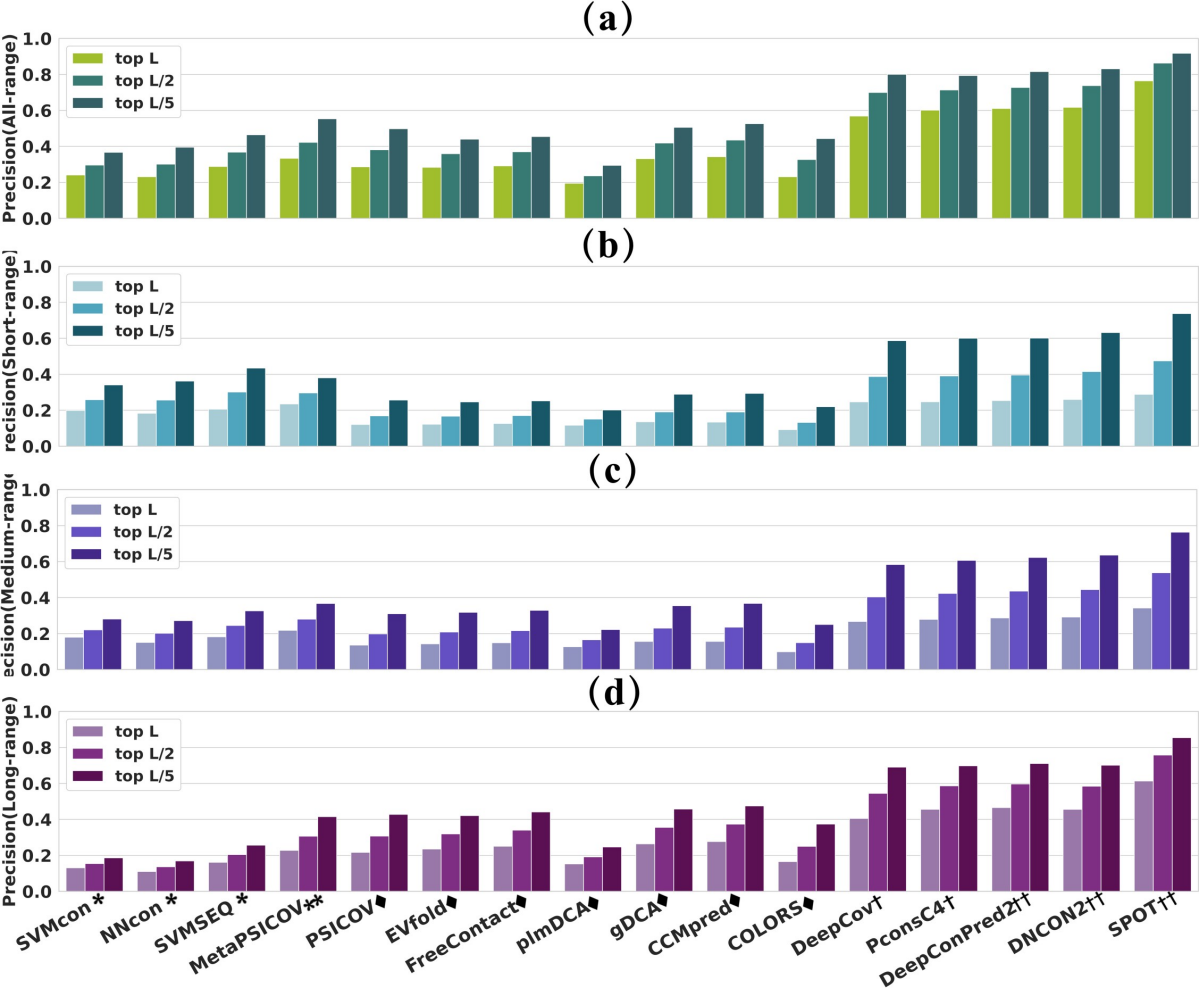
**The overall prediction precision on TestSet1 for (a) short-range (b) medium-range and (c) long-range residue contacts**. Superscripts *, **, ^◆^, ^†^ and ^††^ represent method categories of traditional-ML, consensus-ML, ECA, single-input DL and multi-input DL. DL methods significantly outperform ML and ECA methods. In particular, SPOT achieves the highest precision, outperforming SVMSEQ (the top traditional-ML method), MetaPSICOV (consensus-ML method) and CCMpred (the top ECA method) by 45.3%/59.8%, 36.5%/44.0% and 39.2%/ 38.0% for top L/5 long-/all-range predictions.

The prediction precisions of all-/short-/medium-/long-range contacts for 16 methods on TestSet1 are shown in Fig 1. For short-range contact (that are easier to predict than medium-/long-range contacts) predictions, traditional ML methods outperform the ECA methods in top L, L/2 and L/5 predictions. By contrast, ECA methods show higher precisions than traditional ML methods for long-range contact predictions. The phenomenon could be explained by the following two reasons. Firstly, traditional ML methods and ECA method adopt different prediction strategies. The traditional ML methods are local methods that make a prediction for each residue pair respectively, while the ECA methods are global methods that make predictions by treating correlated pairs of residues as dependent on each other, rather than as statistically independent. Secondly, the distributions of the three contact types (short-/ medium-/ long-range) are different. The number of long-range contacts is much larger than the number of short- and medium-range contacts. The consensus ML method MetaPSICOV, which combines features from both traditional ML and ECA methods, shows close precisions to traditional ML methods for short-range contact prediction and similar precisions to ECA methods for long-range predictions. DL methods, which can capture the higher-order residue correlations and use nonlinear models with fewer parameters to be estimated from thousands of protein families[13], significantly outperform ML and ECA methods. In general, DL methods show the best overall performance with precisions ranging from 57%-76%, 70%-86% and 80%-92% for top L, L/2 and L/5 predictions, respectively. Specifically, SPOT shows prediction precisions significantly higher than other methods.

The prediction precisions of 18 methods on TestSet2 are presented in S1 Fig. Similar to that in Fig 1, the results in S1 Fig also verify the significant performance improvement of DL methods over ML and ECA methods. In particular, DL methods such as SPOT/ trRosetta/ RaptorX that trained on large-scale training sets and high-quality features show the highest prediction precisions. S1 Fig also shows that SPOT, trRosetta and RaptorX have very close precisions in top L/ L/2/ L/5 predictions for all the evaluated sequence separations. Traditional ML methods show almost the same precisions in S1 Fig as that in Fig 1 for short-/ medium- /long-range contact prediction. However, ECA and DL methods exhibit poorer performance in S1 Fig than in Fig 1 for different ranges, especially for the long-range predictions. This is mainly because the average ***N***_***eff***_ in TestSet2 is shallower than that in TestSet1, suggesting that coevolution data could be an important driver for high-quality contact prediction.

The results in both Figs 1 and S1 demonstrate that the evolution of methods promotes the performance of contact prediction: the prediction precision has been improved from about 40% /20% using traditional ML methods to 46%/43% by ECA, 55%/42% by consensus ML and 83%/73% by DL (averaged on all-/long-range top L/5 predictions in Fig 1). The extensively trained DL approaches dominate current algorithms, which are mainly because of the ever-increasing sequence databases, diverse feature types, well-designed networks and the up-to-date large training sets.

### Prediction similarity between different methods

**Fig 2:**
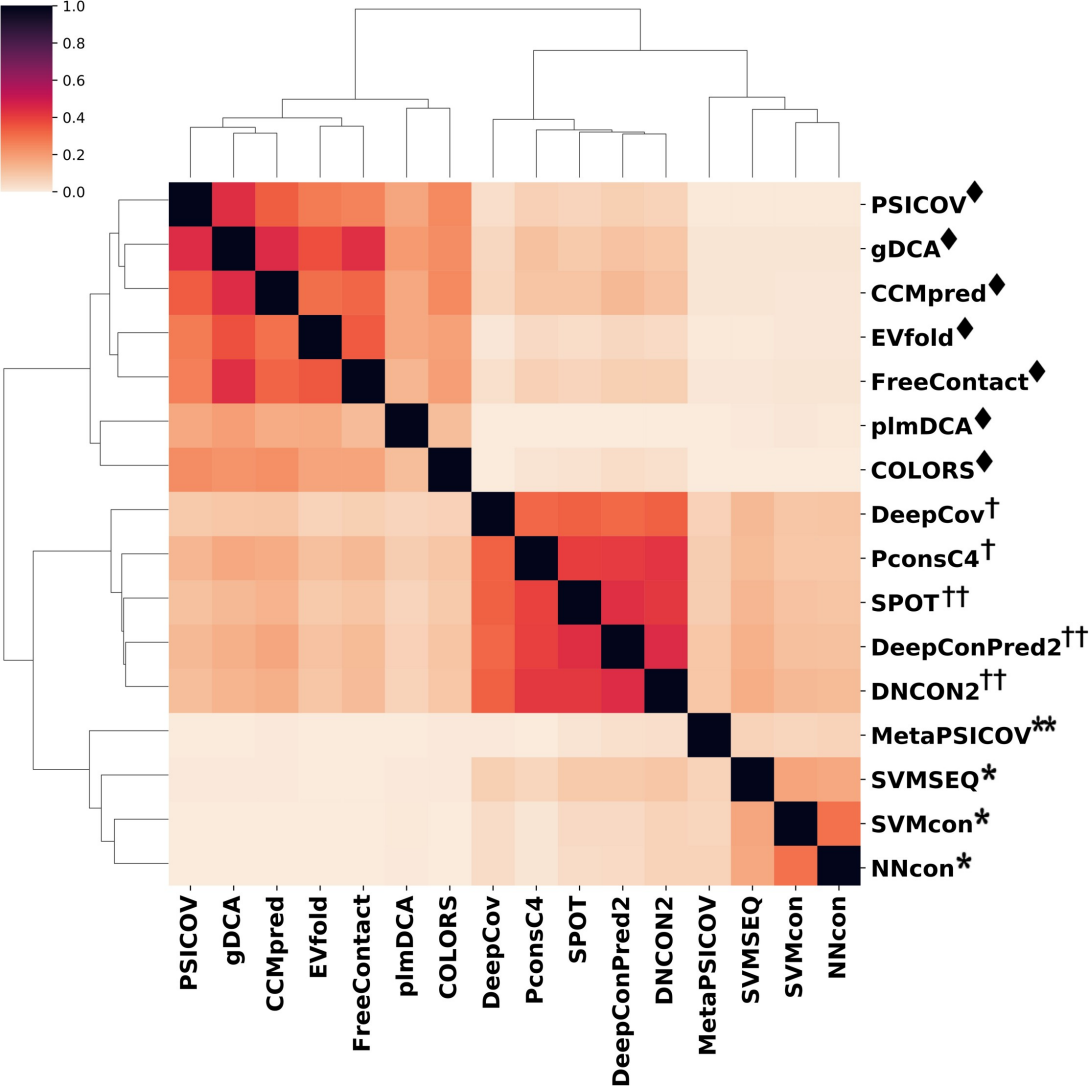
Jaccard index and evolutionary relationship between 16 predictors. The Jaccard indices are calculated using top L predicted contacts for each protein in TestSet1 and then averaged on the whole TestSet1. The clustering results indicate DL methods show stronger prediction similarity between each other than ML and ECA methods.

Evaluation of the prediction similarity between different methods is important for investigating the evolutionary relationship between these methods. Fig 2 shows the dendrogram heatmap of Jaccard indices using Ward’s hierarchical clustering method on TestSet1 (S2 Fig on TestSet2). The Jaccard index between two methods is calculated by averaging the Jaccard index value of each protein on the whole test set. According to the clustering results, these methods can be roughly divided into three categories, namely traditional ML methods, ECA methods and DL methods. Apparently, traditional ML methods are highly divergent from ECA methods, while slightly close to multi-input DL methods. Specifically, PSICOV/ gDCA, FreeContact/ gDCA, gDCA/ CCMpred, PconsC4/ DeepConPred2, PconsC4/ DNCON2, PconsC4/ SPOT, DeepConPred/ DNCON2, DeepConPred2/ SPOT and DNCON2/ SPOT show relatively high Jaccard indices of 0.463, 0.460, 0.469, 0.465, 0.479, 0.455, 0.497, 0.488, 0.469. DL methods show stronger prediction similarity between each other, with average Jaccard indices 0.151 and 0.278 higher than ECA and ML methods.

The similarities of different methods are further investigated in a more detailed manner. In S3 Fig, the studied objects are individual proteins instead of the whole test set, and only proteins with high prediction similarities are shown. For proteins with Jaccard index > 0.5, ML methods show sparse links with other methods while DL methods share highly mutual overlap on many proteins. The prediction results of plmDCA and COLORS are not strongly overlapping with other ECA methods, which is consistent with the clustering results in Fig 2.

The similarity of the prediction results of the DL methods is partially due to the high accuracy of these methods, and on the other hand, because the main inputs/feature types used by their models are very similar (see S1 Table). The Jaccard indices also indicate the evolutionary relationship between these predictors. Traditional ML methods are far from ECA methods since these two categories share not too many similar inputs and features and also differs in prediction strategies (ECA are global prediction methods while ML are local prediction methods, which differs at predicting the contact probability of one residue pair by considering its correlation with other residue pairs or not). Although MetaPSICOV combines several ECA methods, it is encompassed in the cluster of ML methods together with the traditional ML methods. This is probably because: (1) The consensus strategy adopted by MetaPSICOV (MetaPSICOV combines different ECA methods using machine learning techniques). (2) Both MetaPSICOV and traditional ML methods use local prediction strategies. DL methods show close “genetic” relationships with ECA methods probably because almost all DL methods are inspired by the idea of direct coupling analysis and even adopt ECA methods as part of the prediction module.

### Performance in terms of model probability (score)

The probability (score) of a predicted residue pair given by a machine learning/deep learning/ECA model indicates the category (contact/noncontact or distance bins) to which the residue pair is most similar. The probabilities (scores) given by the methods show the confidences of the predictions. Because the predicted contacts are selected by sorting the probability (score) or through a probability (score) threshold, the reliability of the probability (score) can greatly reflect the reliability of the model. The prediction probability (score) ranges for 16 methods on TestSet1 are shown in S2 Table. Rather than evaluating all the methods with the same probability (score) standard, this section focuses on the performance trend of each method as the probability (score) threshold changes.

**Fig 3:**
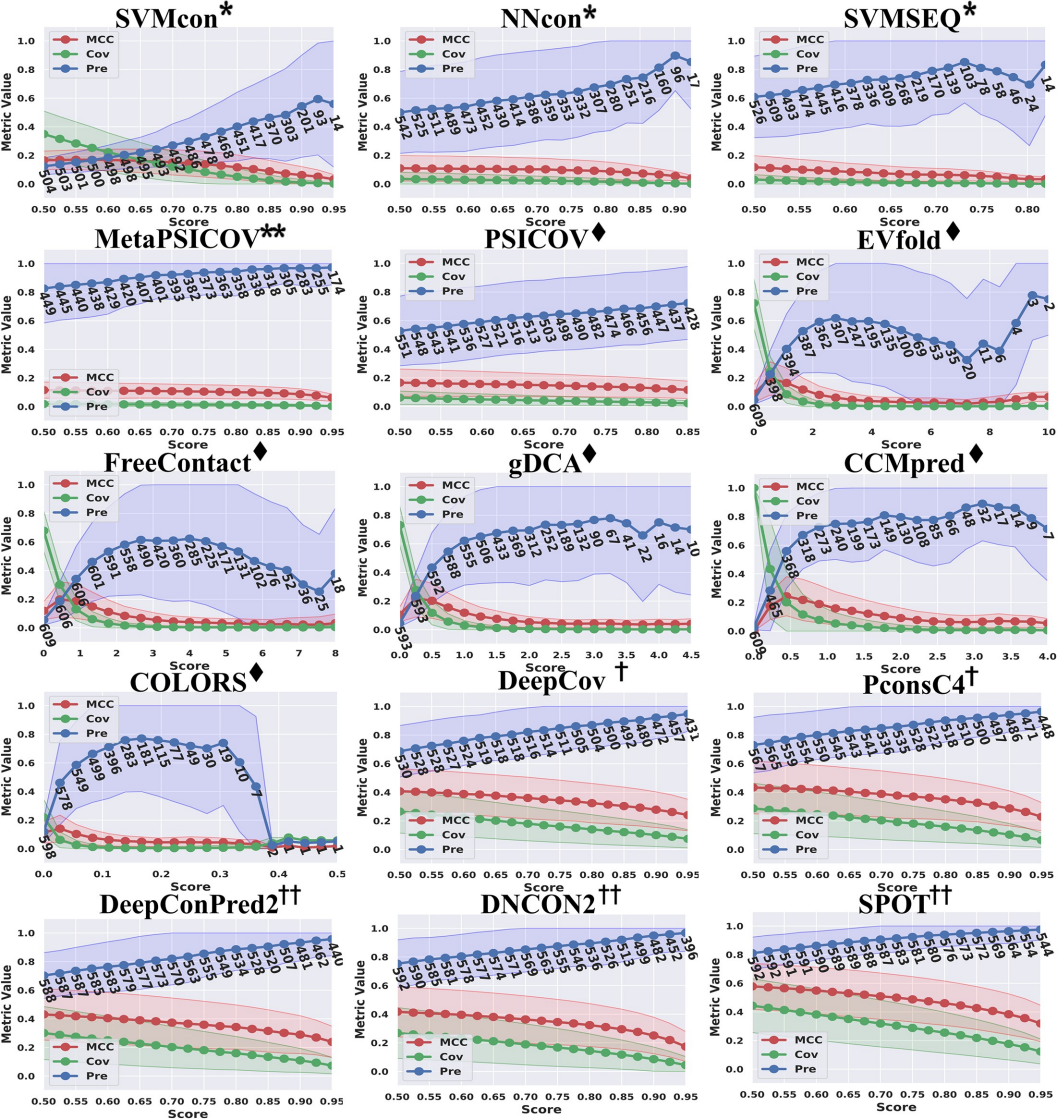
Prediction performance in terms of precision, coverage, MCC and the corresponding standard deviation (the shaded area around the curves) with the increasing probability (score) threshold given by the predictors. For the methods with probability (score) distribution between [0,1], the x-axis uses 0.5 as the starting threshold, and for some ECA methods with wider probability (score) distribution ranges, the x-axis starts at 0. The numbers under the precision curve (blue) are the numbers of proteins with predictions returned using the corresponding probability (score) threshold on the x-axis. DL methods show much higher reliability in model probability (score) compared with ML and ECA methods.

Fig 3 illustrates the prediction performance in terms of different metrics with the increasing probability (score) threshold given by the prediction methods on TestSet1. It is shown that the prediction coverages for all methods decrease gradually with the increase of the probability (score) threshold. The prediction precisions of DL methods (DeepCov/ PconsC4/ DeepConPred2/ DNCON2/ SPOT) increase monotonically with the probability (score) threshold. Nevertheless, this trend is not monotonous for traditional ML methods SVMcon/ SVMSEQ and ECA methods FreeContact/ plmDCA/ gDCA/ COLORS; as the threshold increases, their precision curves go down at some probability (score) value. Traditional ML methods and ECA methods also show much larger STDs on precisions and relatively lower coverages/ MCCs compared with DL methods. Through integrating advantages of both ECA and traditional ML methods, “shallow” neural network-based method MetaPSICOV shows very satisfactory prediction precisions with low STDs and the precision curve is similar to that of the “deep” neural network-based method SPOT. However, MetaPSICOV still cannot overcome the inherent drawbacks of low coverage and MCC, which results in fewer and fewer proteins with predictions returned as the probability (score) threshold increases. On the contrary, DL methods can still maintain a larger number of predicted proteins and MCCs of no less than 0.2 as the probability (score) threshold increases.

Note that there are 10 proteins without any native non-local contacts, and for 9 of them, the multi-input DL method SPOT successfully makes no positive contact prediction (probability < 0.5). For the remaining protein, SPOT only gives two positive predictions with probabilities of 0.50 and 0.58. Other DL methods such as DeepCov, PconsC4, DeepConPred2 and DNCON2 correctly make no positive predictions for 7, 6, 8 and 6 proteins, respectively. The analyses from both the performance curve and the special cases prove that DL methods represented by SPOT are highly reliable in terms of the model’s prediction probability (score).

### The MSA with *N*_*eff*_ > 5L-8L permits “the best for all” and enables “simplicity over complexity”

MSA plays a fundamental role in computational biology for analyzing the functional, structural or evolutionary relationships between a set of protein sequences[65], and also performs as a central component in protein contact prediction because the co-evolutionary coupling signal is the most important contributor to many prediction models. TestSet1 is divided into 7 different groups in terms of *N*_*eff*_, namely, < 5, 5–0.2L, 0.2L-L, L-5L, 5L-8L, 8L-15L and > 15L, where L is the protein sequence length.

**Fig 4:**
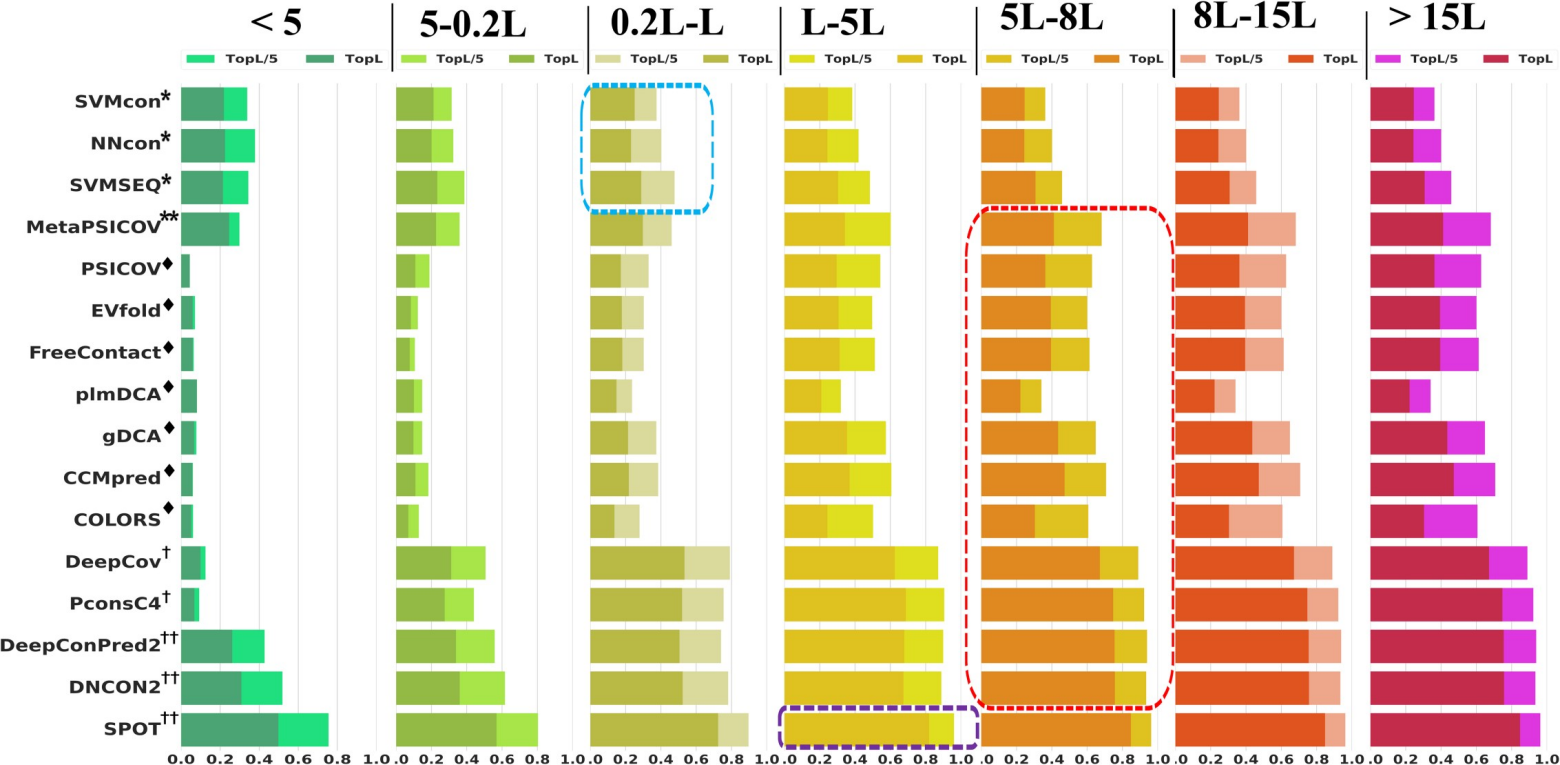
Prediction precisions of top L and L/5 contacts with the variation of *N*_*eff*_ on TestSet1. From left to right the bar plots illustrate the prediction precisions of top L contacts for proteins with *N*_*eff*_ < 5/ 5–0.2L/ 0.2-L/ L-5L/ 5L-8L/ 8L-15L/ > 15L. Methods in the blue/red/purple squares show the best performance with minimum *N*_*eff*_ of 0.2L-L/L-5L/5L-8L. The MSA with *N*_*eff*_ > 5L-8L permits “the best for all” and enables “simplicity over complexity”

As shown in Fig 4, traditional ML methods (SVMcon/ NNcon/ SVMSEQ) are barely affected by the low *N*_*eff*_ and show no precision increase when *N*_*eff*_ > L. All ECA methods and single-input DL methods (DeepCov/ PconsC4) which are based purely on MSA perform poorly for low *N*_*eff*_ proteins. The precisions of consensus ML and multi-input DL methods decrease more slowly than ECA and single-input DL methods. However, these methods still show low prediction precisions for proteins with shallow MSA.

Multi-input DL method SPOT obtains the highest prediction precisions of 81% and 95% for top L and L/5 predictions with *N*_*eff*_ > L-5L, and shows no further improvement as *N*_*eff*_ increases. All ECA methods and most DL methods (DeepCov/ PconsC4/ DeepConPred2/ DNCON2) that rely on MSA show the best performance with *N*_*eff*_ > 5L-8L. One interesting result is that, with enough MSA (*N*_*eff*_ > 5L-8L) as input, some methods with less model complexity or less computational efforts (“simplicity”) show comparable or even better performance than methods trained on large training sets or relying on multi-categories of inputs (“complexity”). For example, single-input DL methods like PconsC4/ DeepCov (“simplicity”) can achieve comparable performance with multi-input DL methods like DeepConPred2/ DNCON2/SPOT (“complexity”) and some ECA methods (“simplicity”) can even outperform the consensus method MetaPSICOV (“complexity”).

Previous studies showed that the features from MSA-related statistics play the most significant role among all feature categories for DL methods[11,50]. In this study, (1) we can see that the quality of MSA can entirely decide the performance of ECA/ single-input DL methods and greatly affect the performance of consensus ML/multi-input DL methods. (2) We also find that with 5L-8L effective sequences in MSA, all methods show the best performance and those methods that rely only on MSA as input can reach comparable achievements as methods that adopt multi-categories of inputs. These findings prompt us to search some large databases (such as the nr database used in this study or metagenomics in [55]) for low-homology protein targets, and use simple methods (such as single-input DL methods) instead of complex methods (such as multi-input DL methods) for proteins with *N*_*eff*_ > 5L.

### Prediction performance in terms of sequence length, contact density and structural class

**Fig 5:**
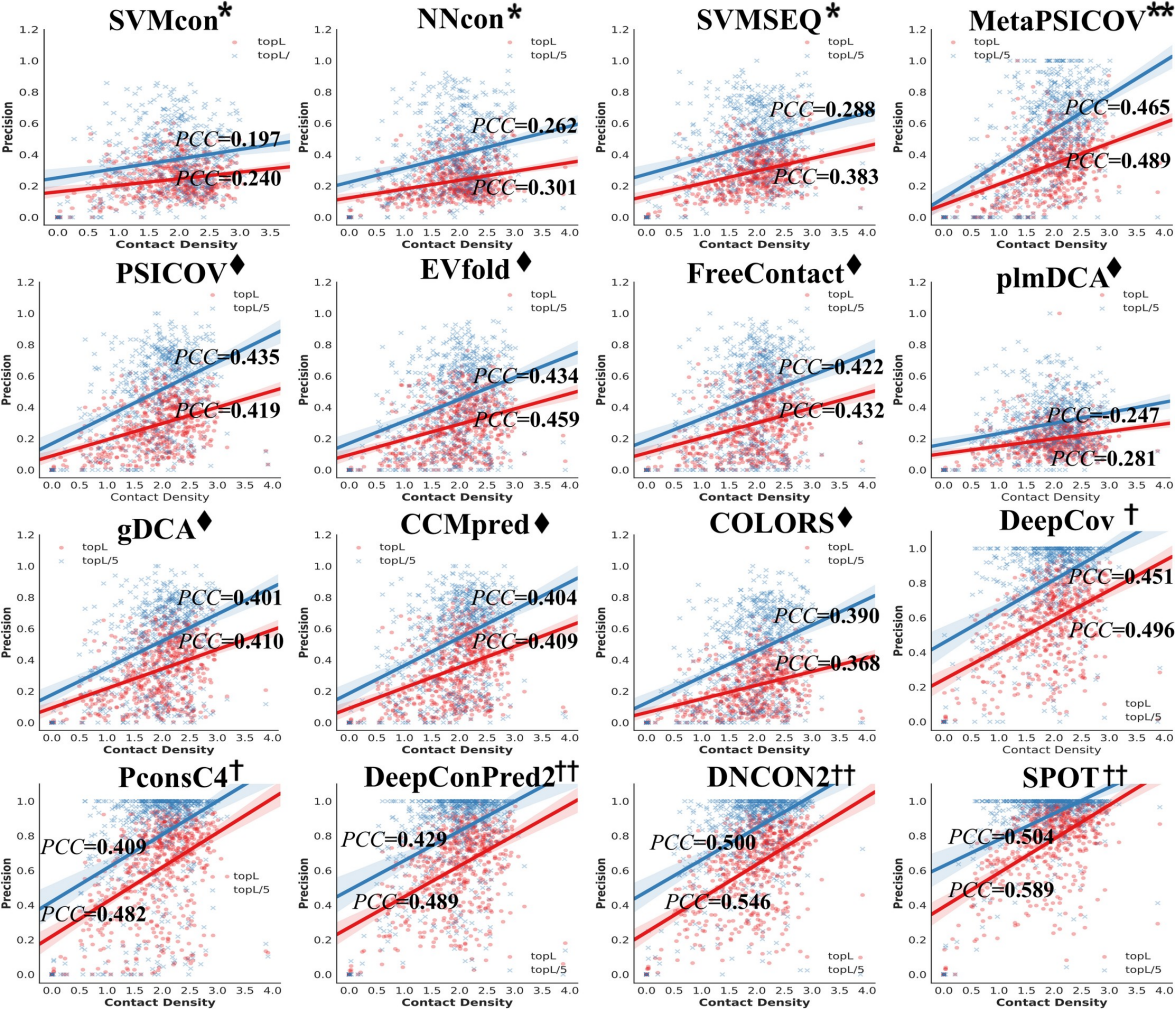
The plot of precisions for all-range contacts versus contact density on TestSet1. Red dots and blue crosses indicate the targets for top L and top L/5 predictions, respectively. Pearson’s correlation coefficient (PCC) is used to measure the linear correlation between prediction precision and contact density. All methods show correlations between precision and contact density.

We first compare the performance upon sequence length (S4 Fig) and contact density (Fig 5) for the 16 methods on TestSet1. Pearson’s correlation coefficient (PCC) is used to measure the strength of the linear correlation between prediction precision and sequence length/contact density. While there is no significant correlation between the sequence length and precision (which is consistent with the previous study[66]), a correlation between precision and contact density can be observed. As demonstrated in [67], the density of contacts is higher in the neighborhood of a contact than in the neighborhood of a non-contact. Contact density impacts prediction precision because many methods especially the DL methods make use of contact occurrence patterns[11,62] for prediction. When contact density is low, it is hard to identify reliable contact patterns. However, prediction precision and contact density are not always positively correlated: through more detailed analysis on proteins with N_eff_ > L, we find that the positive correlation is visible for density values in the range of 0.0–2.0 and strong correlation only lies in proteins with contact density 0.0–1.0 (S3 Table and S5 Fig).

We further analyzed the prediction performance on five different structural classes, ie α, β, α+β (α/β), transmembrane (TM) and proteins with intrinsic disorder regions (IDR), consisting of 71, 54, 364, 9 and 6 single-domain proteins, respectively. Although the numbers of TM and IDR targets are scarce, they are very representative since they are highly non-redundant with other proteins in the test set and training sets of all methods. As tabulated in Table 1, the highest prediction precision for α/ β/ α+β/ TM/ IDR proteins are 55.3%/ 81.6%/ 79.3%/ 78.3%/ 50.7% and 77.4%/ 92.7%/ 93.9%/ 88.2%/ 76.2% for top L and top L/5 predictions, respectively. The results underscore that contacts in β and α+β proteins are the easiest to predict, followed by that in TM proteins, while contact predictions for α and IDR proteins produce the lowest precisions. There may be several reasons for the differences in prediction precision. Here, we try to explain the phenomenon through the contact densities of different protein structural classes by considering the impact of *N*_*eff*_. As it’s shown in Section 3.4, the prediction performance could also be greatly affected by *N*_*eff*_. To present a more rigorous analysis on the connection between contact density and structural class, we conduct another performance comparison on different protein types with *N*_*eff*_ > L (S4 Table). The contact densities for IDR/ α/ TM /α+β /β proteins in Tables 1 and S4 are 0.94/ 0.97/ 1.80/ 1.97/ 2.30 and 0.46/ 1.09/ 1.99/ 2.0/ 2.43, respectively. Through comparing the prediction precisions and contact densities of different structural classes in the same table (either Table 1 or S4 Table), we can get some evidence for our previous conclusions that there is a correlation between precision and contact density for proteins with contact density<2.0 (strong correlation for contact density <1.0) and no positive correlation for proteins with contact density≥2.0.

**Table 1:** Prediction precisions of top L, L/2 and L/5 all-range predictions by 16 methods on different protein types with *N*_*eff*_ > 0

Methods	α (topL)	α (topL/5)	β (topL)	β (topL/5)	α+β (topL)	α+β (topL/5)	TM (topL)	TM (topL/5)	IDR (topL)	IDR (topL/5)
**SVMcon**	18.0%	27.1%	30.4%	40.0%	25.6%	40.1%	19.4%	23.5%	19.2%	34.6%
**NNcon**	12.8%	22.9%	30.3%	46.5%	24.9%	43.1%	12.8%	23.0%	19.8%	25.8%
**SVMSEQ**	17.6%	31.8%	34.1%	46.2%	30.8%	50.1%	24.8%	36.6%	21.1%	37.6%
**MetaPSICOV**	15.0%	21.3%	43.2%	62.6%	35.5%	60.6%	37.3%	46.6%	24.8%	31.9%
**PSICOV**	17.2%	31.3%	28.7%	48.5%	30.2%	52.3%	35.5%	61.6%	18.3%	27.6%
**EVfold**	17.2%	28.6%	28.5%	44.2%	29.9%	45.8%	38.5%	62.5%	19.2%	27.4%
**FreeContact**	17.9%	29.8%	29.2%	44.8%	30.6%	47.3%	38.0%	61.2%	19.2%	27.4%
**plmDCA**	14.0%	22.2%	26.2%	36.5%	19.8%	30.1%	19.3%	30.6%	13.3%	19.1%
**gDCA**	19.4%	32.0%	33.3%	50.0%	34.7%	52.5%	43.6%	67.1%	16.8%	28.4%
**CCMpred**	19.9%	32.5%	32.6%	51.2%	36.0%	54.8%	49.6%	73.9%	18.1%	32.9%
**COLORS**	13.6%	25.8%	24.3%	42.1%	24.0%	46.4%	32.1%	58.5%	9.7%	22.5%
**DeepCov**	36.7%	59.5%	67.9%	83.8%	59.3%	83.3%	63.3%	82.6%	29.3%	39.2%
**PconsC4**	34.9%	53.6%	61.2%	70.5%	63.8%	83.9%	69.6%	86.5%	30.4%	47.2%
**DeepConPred2**	40.6%	63.0%	63.2%	80.5%	63.9%	84.6%	63.3%	74.1%	38.1%	63.9%
**DNCON2**	39.4%	63.2%	65.3%	83.4%	64.8%	86.5%	73.2%	85.6%	40.1%	61.6%
**SPOT**	55.3%	77.4%	81.6%	92.7%	79.3%	93.9%	78.3%	88.2%	50.7%	76.2%

*Noted*: There are 71, 54, 364, 9 and 6 single-domain proteins in the test set of α, β, α+β (α/β), transmembrane (TM) and proteins with intrinsic disorder regions (IDR).

The “natural” difficulty of contact prediction in the low-contact-density proteins encourages the exploration of corresponding coping strategies: (1) only a limited number of top predictions are recommended to avoid the high risk of low precision; (2) referencing the predicted probability given by DL models can further improve the credibility of the results. A successful story can be found in S5 Table. Through considering both top L/*n* and model probability strategies, almost all DL methods show improvements in precisions for the IDR proteins. Specifically, SPOT can get a satisfactory precision of 91.5% with only a small increase in the probability threshold to 0.6, and also rules out predictions for a highly intrinsically disordered protein that contains no native contacts.

### Prediction performance on physicochemical interactions

Protein tertiary structure is the spatial arrangement of each secondary structure and is stabilized by physicochemical interactions such as hydrogen bonding, salt bridges, disulfide bonds, and non-polar hydrophobic interactions. The connection between the physicochemical nature and the predicted contacts derived from evolutionary information may be highly relevant to protein structure and function prediction. In this section, physicochemical interactions of contacts driven by hydrophobic interaction (HB, defined as CB-CB with a cut-off distance of 5Å), salt bridge (SB, formed between any of the oxygen atoms of acidic residues and the nitrogen atoms of basic residues within a cut-off distance of 3.2 Å) and disulfide bond (DB, formed between two sulfur atoms within the distance of 2.05 ± 0.03Å) are used for evaluation. Since the residue contact predictors are not specifically designed for the physicochemical interactions, we will not evaluate their prediction precision but focus on the coverage.

**Fig 6:**
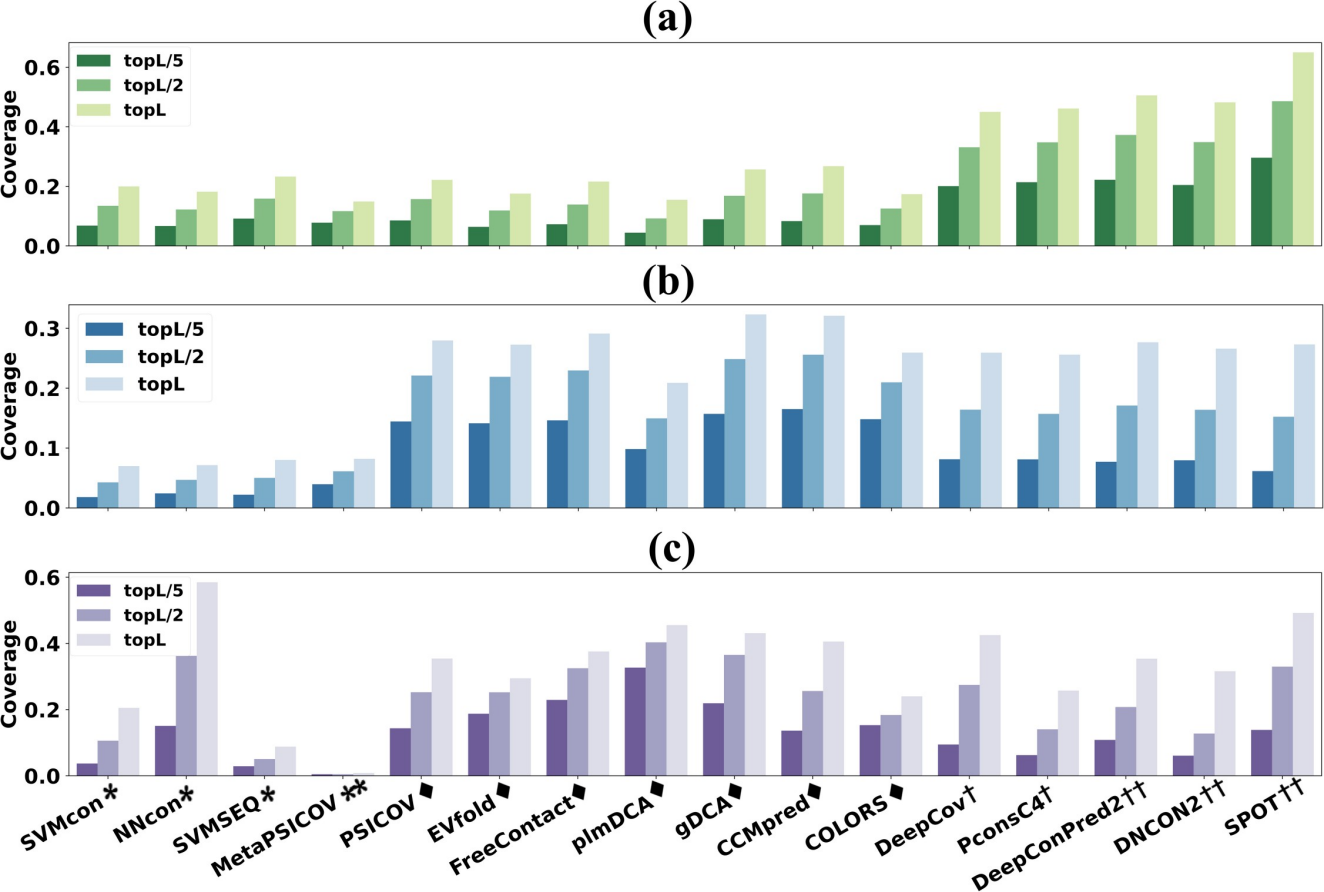
**Prediction coverages of 16 methods on TestSet1 for physicochemical interactions:** (a) hydrophobic interactions (b) salt bridges (c) disulfide bridges. For top L/5 predictions, DL methods can predict more hydrophobic interactions while ECA methods predict more salt bridges and disulfide bonds.

Fig 6 shows the prediction coverages of hydrophobic interactions, salt bridges and disulfide bridges on TestSet1 (S6 Fig on TestSet2). The amount of physicochemical interactions for a specific category is much less than the number of residue contacts in a protein, so the analyses are mainly based on the top L/5 contact predictions. The prediction trend of HB in Fig 6A is similar to that of the contact prediction, with multi-input DL methods (DeepConPred2/ DNCON2/ SPOT) in the leading positions and closely followed by the single-input DL methods (PconsC4/ DeepCov). DL methods show average coverage of 22.7% for top L/5 predictions, which is 15.1%/15.4% higher than ML/ECA methods. Interestingly, ECA methods outperform all the DL methods in SB (Fig 6B) and DB (Fig 6C) with average coverages of 14.3% and 19.9% for top L/5 predicted contacts, which are 11.7%/ 7% and 14.4%/ 10% higher than ML/DL methods. Drawn from results for 18 methods on TestSet2 (S6 Fig), the conclusion that DL methods can predict more hydrophobic interactions while ECA methods predict more salt bridges and disulfide bonds (for top L/5 predictions) is similar to that for 16 methods on TestSet1(Fig 6).

**Fig 7:**
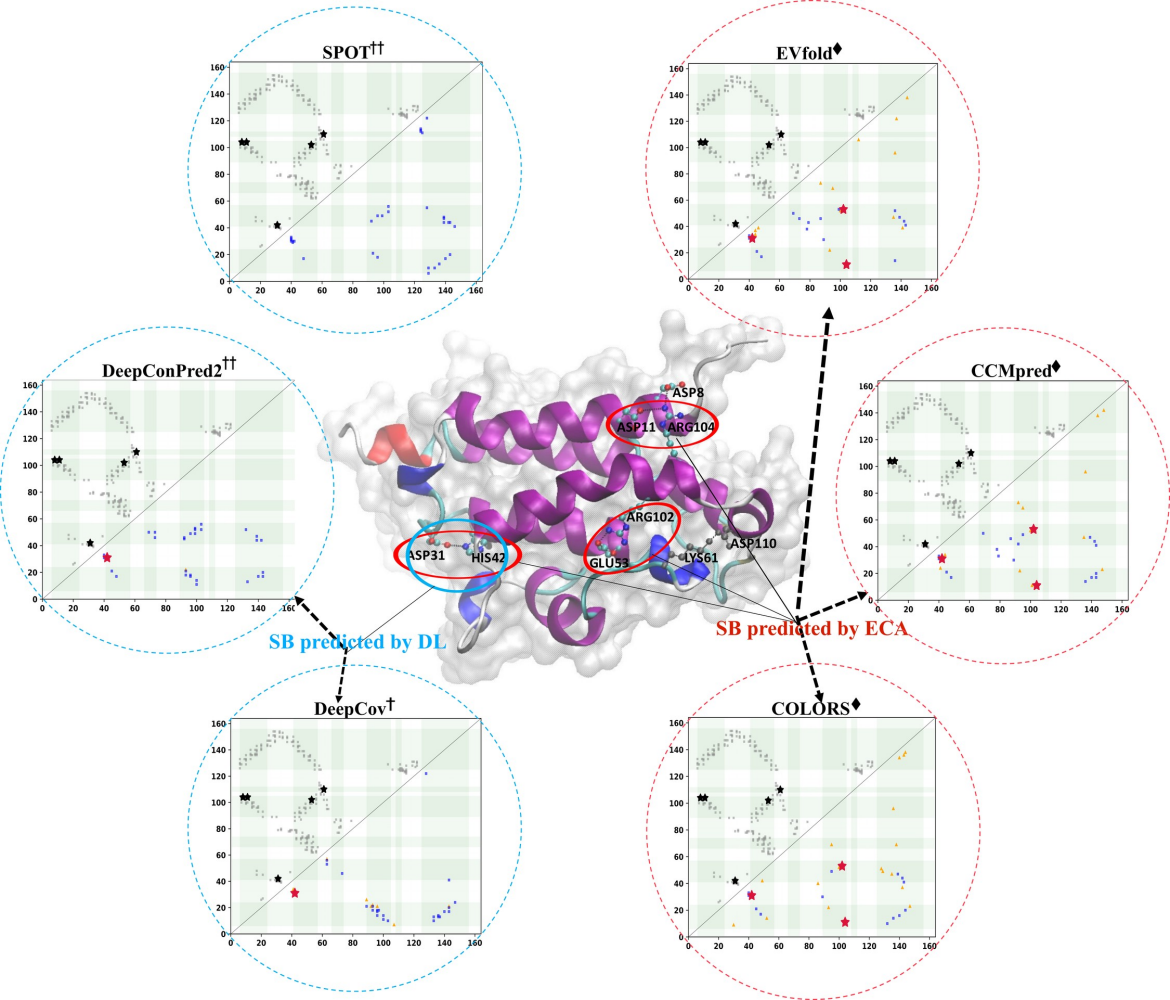
The predicted contact maps with salt bridges for 5WK0A (top L/5 predictions with a separation of no less than 6 residues). The cartoon at the center illustrates the salt bridges in the 3D structure. The gray square/blue square/orange triangle and black/crimson star stand for the native /true positive/ false positive contact and native/predicted salt bridge, respectively. ECA methods can detect more contacts related to salt bridges than ML and DL methods.

A demonstrative example (PDB ID: 5WK0A, which is the bacillithiol transferase BstA with a sequence length of 163 and *N*_*eff*_ of 829) shows the contact prediction with salt bridges for top L/5 predictions by different methods (Fig 7 and S6 Table). The average coverage of SB for top L/5 predictions is 0/ 52%/ 8% for ML/ ECA/ DL methods. Although DL method SPOT can achieve 100% precision for top L/5 contact predictions, it predicts no SB at all. By Contrast, ECA method COLORS shows a contact precision of only 42.7%, but obtains a coverage of 60% for SB prediction. Fig 7 also underscore similar prediction patterns for methods in the same category and the connection between the physicochemical interactions and the co-evolutionary couplings from the MSA.

The results suggest residue contacts related to physicochemical properties can be detected from current contact predictors. These interactions usually play a very important role in maintaining structural stability, and therefore can often be found in co-evolution. DL methods excel in hydrophobic interaction prediction mainly because hydrophobic interaction is a stricter contact definition between hydrophobic residues, which also explains why residues buried in the core of protein structure are more prone to be in contact than residues on the surface. ECA methods show overall higher prediction coverages for salt bridges and disulfide bonds (in top L/5 predictions) than DL methods, which suggests that the push for higher contact prediction precisions of DL methods might lead to the loss of contacts with some important physicochemical interactions. Possible suggestions for future development are: consider more physicochemical-related properties for model training or develop specific predictors for contacts of physicochemical interactions.

### Secondary structure related prediction performance

The tertiary fold of a protein is determined by the packing of secondary structural segments, and the interactions between residue pairs on different secondary structural segments are reflected as non-local contacts on the 2D map.

**Fig 8:**
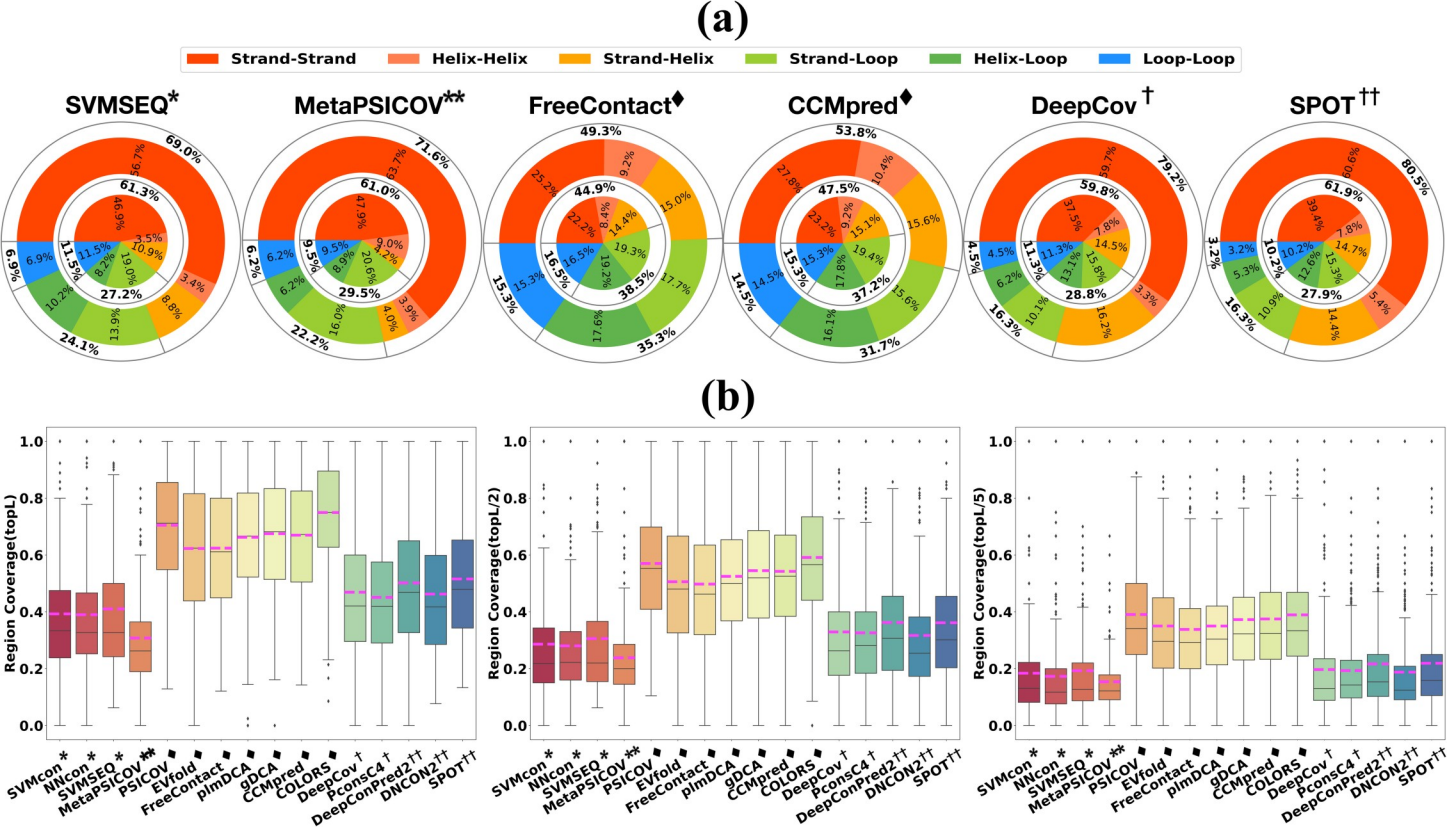
Secondary-structure-related prediction performance on TestSet1. (a) Prediction proportions of true positive contacts for different secondary structure types. The internal pie and external ring and show the proportions of strand-strand, helix-helix, strand-helix, strand-loop, helix-loop, loop-loop in top L and L/5 true positive predicted contacts, respectively. As the number of predictions decreases, supervised techniques are more inclined to predict higher ratios of strand-strand contact types. (b) The box plots from left to right show the prediction region (secondary structure interaction) coverage of top L, L/2 and L/5, respectively (the pink dashed line is the average prediction coverage of each method). DL methods makes use of contact occurrence patterns for accurate prediction, thus the prediction is less dispersed on secondary-structure-based regions when a limited number of top predictions are considered.

To analyze the distributions between different secondary structures of the predicted contacts, we define 6 types of contacts between different secondary structures, namely, Strand-Strand/ Helix-Helix/ Strand-Helix/ Strand-Loop/ Helix-Loop/ Loop-Loop. Figs 8A and S7 show the proportions of 6 contact types in top L and L/5 true positive predicted contacts on TestSet1 (S8 Fig on TestSet2). When compared with ECA methods, both traditional ML and DL methods show much higher prediction proportions of strand-strand contact type but lower proportions of helix-helix/helix-loop/loop-loop types for top L predictions. Notably, the effect is even more pronounced for top L/5 predictions. If we denote the stable secondary structure of helix and strand as HS (H-helix and S-strand), we can find that the prediction proportions of HS-HS, HS-Loop and Loop-Loop in ECA methods are more balanced. The results in S8A Fig for 18 methods on TestSet2 show similar prediction distributions between different secondary structure types. The interaction patterns between different secondary structure types vary significantly. In particular, β-sheets in a contact map are more easily recognizable for supervised ML/DL techniques, which might be caused by the dense visual patterns of β-sheets (the contact density is higher for β-sheet regions compared with other secondary structure types). Another reason could be that contacts between β-sheets (Strand-Strand) are primarily mediated through backbone hydrogen bonds, suggesting that these might not be under the same co-evolutionary pressure as contacts mediated by sidechains[67] (ECA methods are based on co-variation of the sidechains).

We also divide the entire protein structure into more fine-grained regions according to the secondary structures. Each region is centered with a helix or strand, and the two ends of each region are made up of loops. The region-region interaction (secondary structure interaction) coverage is calculated using Eq 3. Two regions are defined as interacting if there is at least one pair of true contact between the two regions. As shown in both Figs 8B and S8B, for top L/n (n = 1, 2, 5) predictions, ECA methods show overall higher coverages of region-region interaction (secondary structure interaction) than DL methods. However, considering the high prediction precisions on contact prediction of DL methods, the reasons behind this phenomenon should be further discussed. Protein contact maps are not random graphs, they are locally structured, and these local structures can be exploited for contact prediction. Therefore, the distribution of contacts follows characteristic patterns and recurring ones can be visually recognized and used for constraining the prediction of nearby contacts[67]. Typical contact patterns on contact maps are shown as clusters correspond to secondary structures. DL methods make use of these contact patterns [11,62] for accurate prediction, so DL methods can accurately excavate more contact patterns (as clusters) and prune isolated false positives.

**Fig 9:**
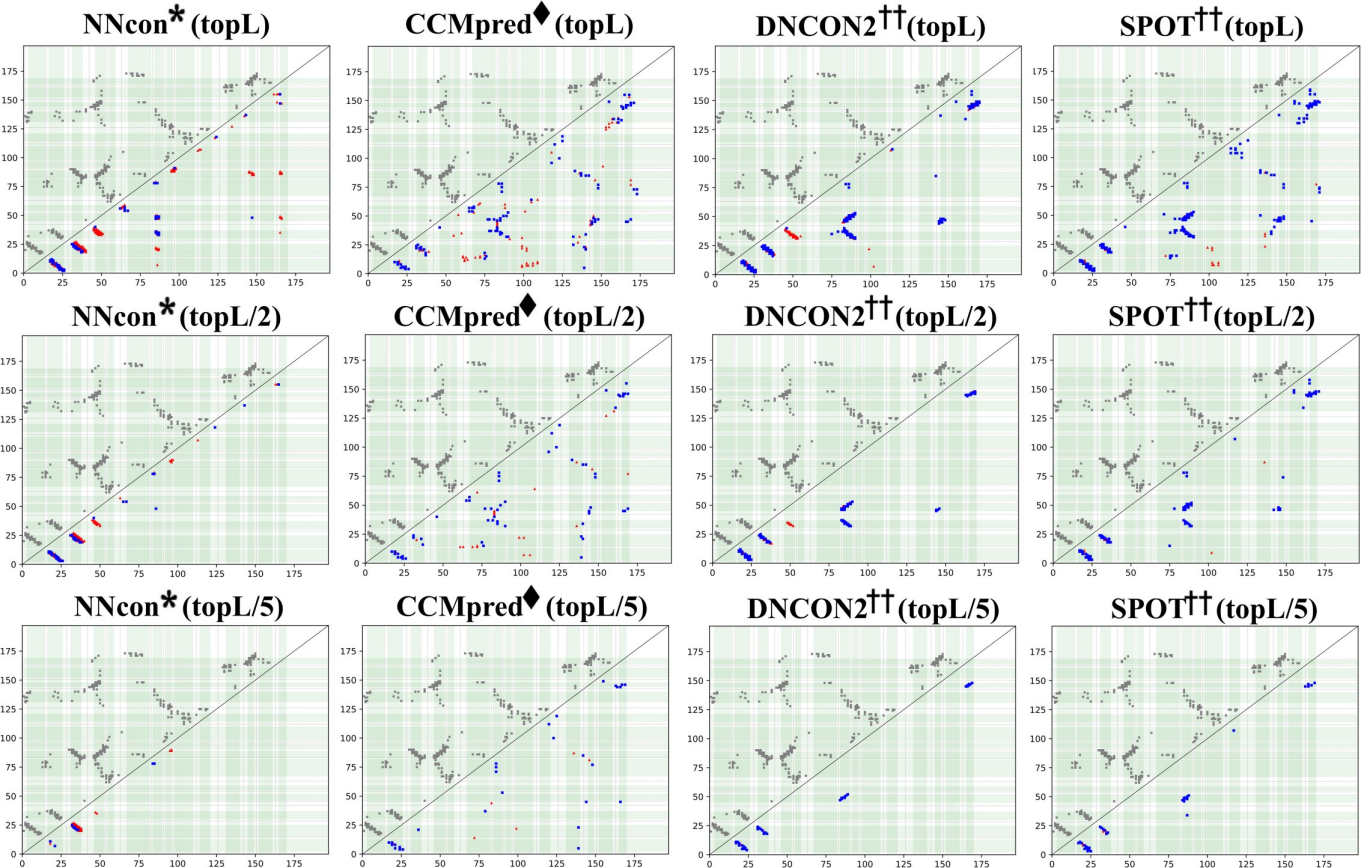
Residue contact map of 2BZ1A by NNcon/ CCMpred/ DNCON2/ SPOT for top L, L/2 and L/5 predictions. Gray squares in the left upper part are the contacts in native structures, blue squares in the right lower part is the correctly predicted contacts and red triangles are wrongly predicted contacts. Regions are divided by the black dashed lines centered with light green secondary structures. When a limited number of top predictions are considered, the predictions by DL methods cover fewer regions, but are more accurate in overall performance and contact pattern recognition.

A representative example (PDB ID: 2BZ1A) in Figs 9 and S9 provides some supports for the above viewpoints. The true positives in top L/n predictions by ECA methods (CCMpred/ gDCA) are more dispersed along the sequence than that by DL methods (SPOT/ trRosetta/ RaptorX), resulting in higher prediction coverages for secondary structure interactions. However, we face a trade-off between wide dispersion and low precision of the predicted contacts when using ECA methods. ML methods (NNcon/ SVMSEQ) and DL methods are more inclined to predict contact as patterns. However, the contact prediction precisions by traditional ML methods are quite low. Unlike ML and ECA methods, DL methods show the more powerful ability for accurate contact pattern recognition while pruning isolated false positives.

### Prediction performance for multi-domain proteins

Multi-domain proteins have many advantages with respect to stability and folding inside cells. Protein evolved through the shuffling of functional domains, and therefore domain-domain interactions from residue contacts are also essential points in 3D structure predictions. Previous studies show that constructing MSAs for individual domains can often improve the intra-domain contact prediction accuracy compared with the full-length sequence[68–70]. Muscat et al. proposed FilterDCA[71], a coevolution-based but structurally supervised contact prediction method, to improve DCA-based contact prediction between interacting domains. Cong et al. applied DCA and GREMLIN in combination with structure modeling to predict protein-protein interactions[21], which can be viewed as an extension application of domain-domain interactions. To evaluate the performance of different methods on multi-domain proteins, we use PDBPfam [72] to identify the domain regions in our dataset. For TestSet1 and TestSet2, there are 108 and 26 protein chains (details in S7 Table) with multiple domains. In this section, we mainly evaluate two aspects for contact prediction of multi-domain proteins: (1) the performance comparison between intra- and inter-domain contact predictions; (2) the prediction balance on different intra-domains in the same protein.

For 108 multi-domain proteins in TestSet1, the prediction results are tabulated in Table 2, and DL methods still hold the leading positions in prediction precisions for intra-domain contacts. Particularly, SPOT achieves a precision of 93.6% for intra-domain contacts in top L/5 predictions. We further analyze the performance between intra- and inter- domains. We can see that ML and DL methods show 18%-32% and 37%-46% higher prediction precisions on intra-domain than that on inter-domain, while the precision differences between intra- and inter-domains are only 5%-17% for ECA methods. Generally, it is more difficult for most methods to accurately predict contacts in inter-domains than in intra-domains, but the prediction difference shown by ECA method is smaller than that of DL and ML. Current methods show lower prediction precisions for inter-domain compared with intra-domain contact predictions, which is true for 18 methods on TestSet2 (S8 Table) as well as for 16 methods on TestSet1(Table 2).

**Table 2:** Intra- and inter-domain prediction precisions of top L, L/2 and L/5 all-range predictions by 16 methods on TestSet1.

Methods	Intra-Domain (topL)	Intra-Domain (topL/2)	Intra-Domain (topL/5)	Inter-Domain (topL)	Inter-Domain (topL/2)	Inter-Domain (topL/5)
**SVMcon**	22.0% (94/108)	25.5% (94/108)	31.2% (93/108)	8.9% (71/84)	12.4% (63/84)	13.3% (43/84)
**NNcon**	26.1% (108/108)	31.4% (107/108)	39.2% (106/108)	8.9% (80/84)	11.0% (70/84)	10.0% (39/84)
**SVMSEQ**	30.2% (106/108)	36.8% (106/108)	45.3% (105/108)	12.6% (81/84)	13.5% (58/84)	12.1% (37/84)
**MetaPSICOV**	35.2% (106/108)	41.9% (105/108)	54.7% (105/108)	20.4% (64/84)	25.5% (53/84)	25.9% (37/84)
**PSICOV**	31.8% (103/108)	42.1% (102/108)	53.4% (99/108)	22.1% (85/84)	31.2% (82/84)	37.0% (71/84)
**EVfold**	20.7% (84/108)	27.7% (83/108)	34.4% (80/108)	16.1% (50/84)	17.8% (44/84)	19.0% (39/84)
**FreeContact**	33.7% (108/108)	42.3% (108/108)	50.7% (105/108)	36.2% (82/84)	41.7% (79/84)	45.5% (72/84)
**plmDCA**	16.8% (75/108)	19.1% (73/108)	24.0% (71/108)	9.3% (57/84)	10.7% (50/84)	15.0% (44/84)
**gDCA**	38.8% (106/108)	49.5% (105/108)	56.9% (104/108)	34.1% (84/84)	40.3% (77/84)	41.9% (69/84)
**CCMpred**	40.4% (108/108)	51.7% (106/108)	59.7% (105/108)	37.4% (82/84)	41.4% (75/84)	42.9% (68/84)
**COLORS**	30.5% (105/108)	41.1% (103/108)	50.3% (100/108)	16.2% (87/84)	22.3% (81/84)	34.6% (71/84)
**DeepCov**	53.6% (94/108)	65.3% (94/108)	71.6% (91/108)	34.7% (72/84)	36.2% (57/84)	32.5% (38/84)
**PconsC4**	66.4% (108/108)	78.8% (108/108)	83.1% (103/108)	48.2% (80/84)	53.0% (64/84)	43.5% (43/84)
**DeepConPred2**	65.5% (108/108)	77.4% (108/108)	83.4% (104/108)	52.8% (77/84)	52.9% (63/84)	46.5% (44/84)
**DNCON2**	65.2% (106/108)	75.3% (105/108)	83.7% (105/108)	53.2% (72/84)	56.3% (62/84)	39.7% (40/84)
**SPOT**	80.7% (108/108)	89.4% (107/108)	93.6% (106/108)	67.0% (71/84)	68.7% (63/84)	47.0% (42/84)

*Noted*: Numbers in parentheses indicate the number of proteins with predicted contacts and the number of proteins with native contacts in corresponding domains.

**Fig 10:**
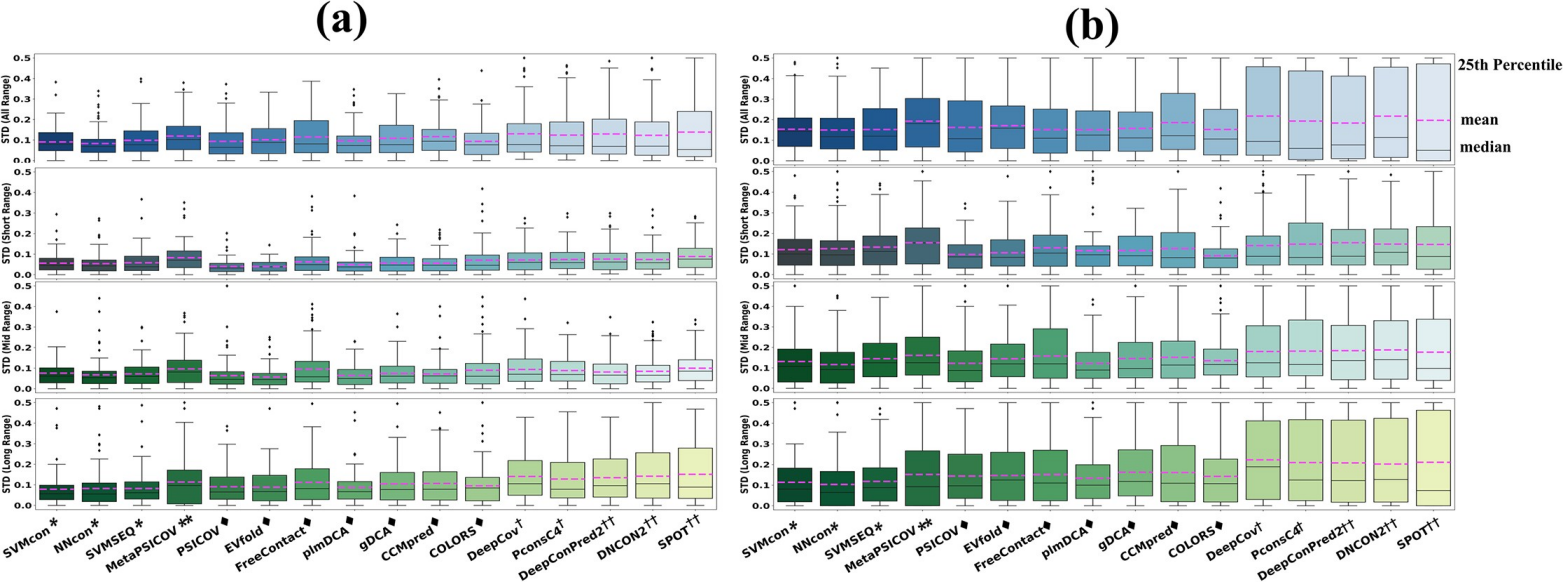
S**TD of prediction precisions between intra-domains in the same protein** in terms of (a) top L and (b) top L/5 predictions. There exist imbalances for top L/5 prediction precisions between intra-domains.

Although all the methods in Fig 10A do not show significant precision differences between different intra-domains within the same protein for top L predictions, however, the STDs in Fig 10B indicate that there exist imbalances for top L/5 prediction precisions between intra-domains, particularly for all- and long-range contacts by DL methods. In top L/5 predictions, all DL methods show precision biases between intra-domains on at least 25% proteins with STD larger than 0.4. Take SPOT for example, there are 25% proteins with STD > 0.45 for all- and long-range contacts, which means for one (some) domain(s) in these proteins the prediction precision(s) is (are) perfectly close to 100%, while the prediction precisions of other domain(s) is (are) close to 0.

The results deliver the facts that precision imbalances exist in (1) inter- *vs*. intra-domains predictions as well as (2) intra- *vs*. intra-domains in the same protein, and the phenomena are more pronounced for DL methods as *n* (in top L/*n*) increases. The results also indicate there are still much room left for improvement in inter-domain or inter-protein contact predictions.

### Runtime comparison of DL methods

**Fig 11:**
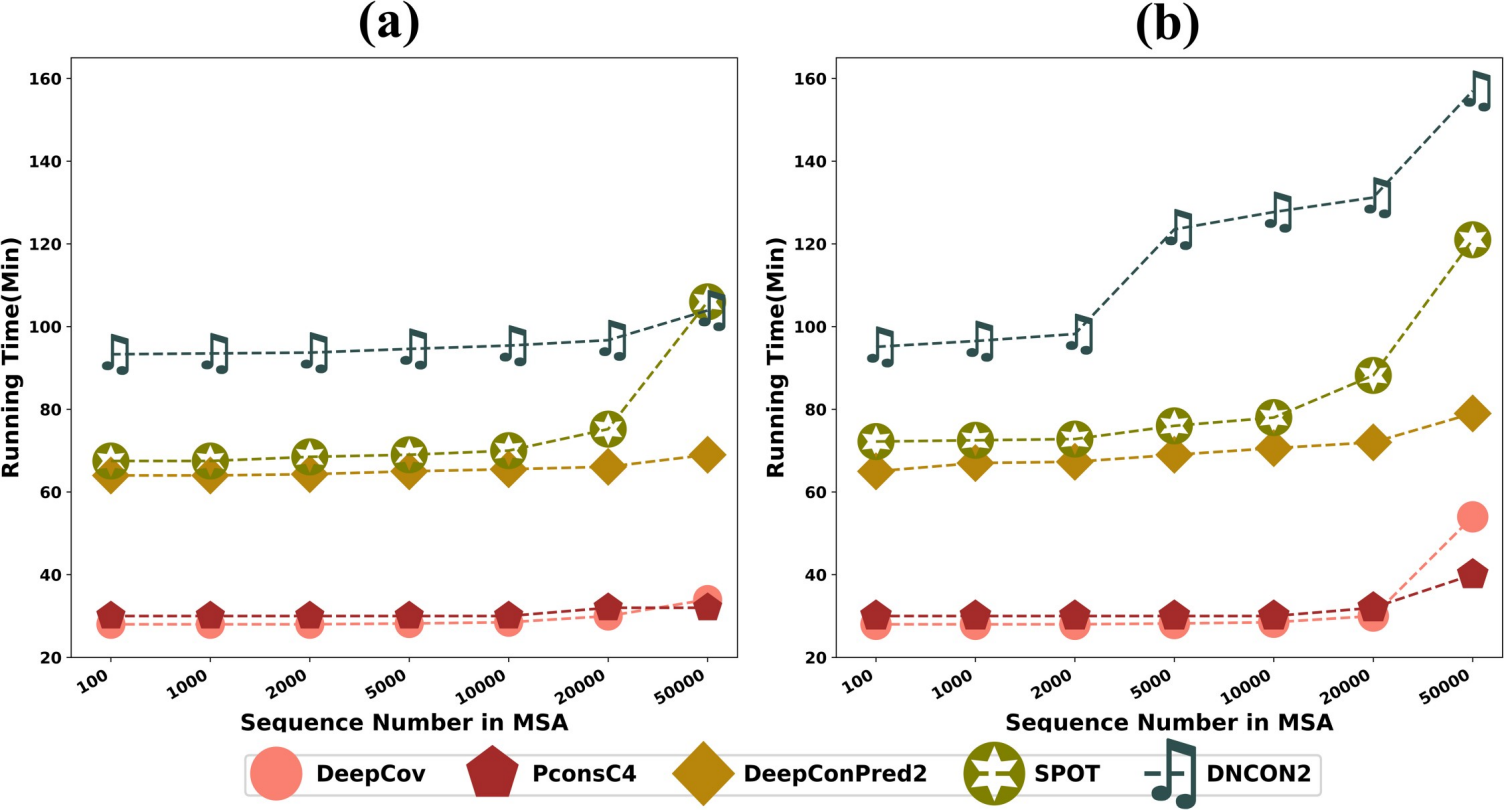
**Runtime tests on proteins with (a) ~200 residues and (b) ~500 residues with the variation of the sequence number in the MSA**.

Since DL methods share many similar inputs as ML and ECA methods (S1 Table), we compare only the runtime of 5 DL methods in this section (Fig 11). The runtime tests are conducted with 24 threads where parallel computing is allowed on Intel Xeon Gold 6230 CPU (2.10GHz) and TeslaV100. We use JackHMMER to search against the nr database for MSA generation. Benchmark proteins with ~200 and ~500 residues are used as the study objects, as shown in Fig 11A and 11B respectively. For the same protein, we also adjust the number of sequences in MSA to observe the robustness of different methods on input data size. It is clear that the more input data type the model requires, the greater the computational cost of the model. When the nr database is used for MSA generation, the runtime of the multi-input method DNCON2 is 3–4 times that of the single-input method PconsC4. If smaller databases are used for MSA search, the runtime benefits of single-input DL methods will be even more prominent. The curves in Fig 11 also indicate that single-input DL methods are more robust in input MSA size.

The execution time for current DL methods is mainly stuck in the feature preparation process rather than the model prediction stage. The typical time-consuming processes include but are not limited to: the PSSM generation process by PSI-BLAST (used by DeepConPred2/DNCON2/SPOT), MSA-based covariance information extraction (used by DeepCov/DNCON2), integration of other contact prediction methods (used by DeepConPred2/DNCON2/SPOT). Possible optimization strategies for development can be (1): PSSM from PSI-BLAST is necessary for proteins with shallow MSA, but may be excluded if a deep MSA (ie. *N*_*eff*_ > 5L) can be obtained. (2) covariance information can be calculated in parallel mode since its time complexity is *pq*^*2*^ (*p* is the height and *q* is the width of MSA). (3) MSA filter can be added before feature extraction to prevent excessive MSA generated by a large database specified by the user.

### Performance of 16 methods on CASP13 targets

32 CASP13 domains are used for evaluation in this section. For blind prediction, the nr database used for generating PSSM and MSA is released before CASP13.

**Fig 12:**
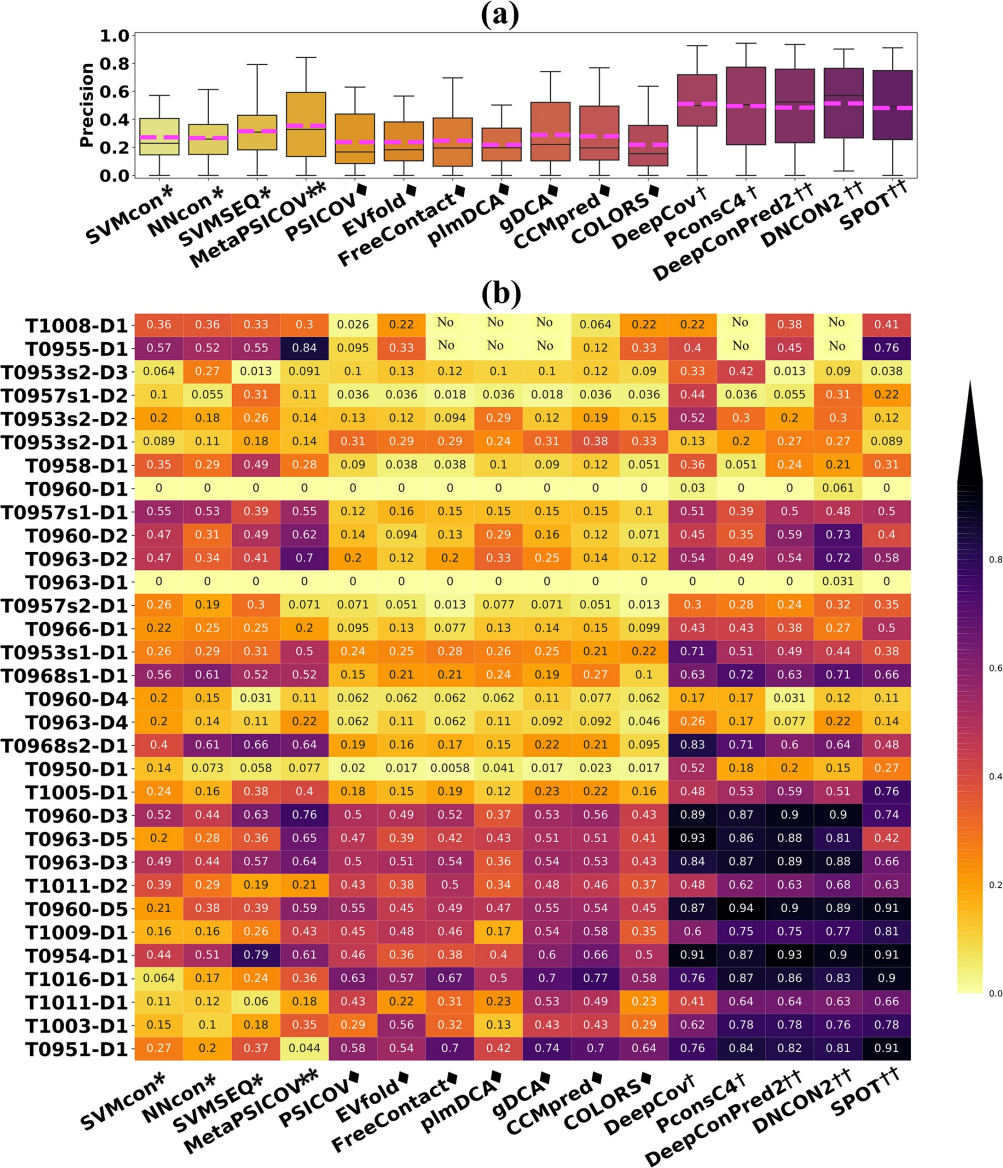
Prediction results for top L predictions of 32 CASP13 domains with sequence separation larger than 6. (a) The box plot of the overall prediction performance. The average prediction precision for each method is labeled with the pink dashed line. (b)The prediction precision distributions for each target of 16 methods. The numbers in the figure represent prediction precisions, and the “No” label indicates that the corresponding method does not return results for the target due to a lack of effective sequences in the MSA.

For average prediction precision in Fig 12A, DeepCov achieves the highest precision of 52.4% for top L predictions, followed by DNCON2 of 50.1%, PconsC4 of 49.5% and SPOT of 48.1%. ML methods and ECA methods perform slightly poorer with precision ranged from and 20%-36% and 22%-29%, respectively. The targets in Fig 12B are arranged from top to bottom in ascending order according to the *N*_*eff*_ of MSA. Although DL methods can achieve very satisfactory results on average, we can see from Fig 12B that there are still exceptions. For some targets with shallow MSAs such as T0955-D1/T1008-D1, SVMcon, NNcon, SVSEQ and MetaPSICOV achieve prediction precisions of 36%/36%/33%/30% and 57%/52%/55%/84%, outperforming DL methods like DeepCov and PconsC4. The performance of all ECA methods and DL methods like DeepCov and PconsC4 largely depends on the number of effective MSA. If shallow MSAs are provided as input, their predictions will fail or perform poorly. For T0960-D1 and T0963-D1 with 2 and 1 native contact(s), the prediction precisions of all methods are 0s. ML methods such as NNcon/SVMSEQ/MetaPSICOV and all DL methods show prediction probabilities <0.308 and <0.583, respectively (S9 Table). Therefore, for proteins with sparse native contacts, instead of trust only the top L/*n* predictions, we also need to consider the prediction probabilities for more accurate analysis.

### Application: contact-guided 3D structure reconstruction

One of the important applications of residue-residue contact prediction is to assist protein tertiary structure reconstruction. In this section, we select CONFOLD2 from the MULTICOM toolbox to conduct the contact-assisted protein folding evaluations. 8 contact prediction methods (traditional ML: SVMSEQ; consensus ML: MetaPSICOV; ECA: EVfold, CCMpred; DL: DeepCov, PconsC4, DNCON2, SPOT) have been used as representatives for testing. Contact-based (instead of distance-based) protein folding are adopted in this study mainly because the development from traditional ML, ECA, consensus ML to DL methods should be viewed as a whole process and be evaluated under the same folding criteria. 251 proteins are selected from TestSet1: 156 proteins with Neff >5L (denoted as TestSet1_Tertiary1) and 95 proteins with Neff <0.2L (denoted as TestSet1_Tertiary2). TestSet1_Tertiary1 consists of 5 TM, 3 IDR, 28 α-rich, 26 β-rich, 44 multi-domain and 50 randomly selected α+β (from 175 α+β proteins with Neff >5L) proteins, while TestSet1_Tertiary2 consists of 1 TM, 2 IDR, 13 α-rich, 12 β-rich, 52 α+β, 15 multi-domain proteins. The results presented in this section are based on the mean TM-score of the best-of-top-five structures and the precisions of predicted contacts averaged on each tested protein.

We first investigate among top L, L/2 and L/5 predictions, which one is most correlated with the quality of the reconstructed structures. Results based on the whole test sets for both proteins with high-quality MSAs (S10 Table) and proteins with low-quality MSAs (S11 Table) indicate that top L predictions are most correlated with the quality of final protein models. For example, in S10 Table, contact prediction based on top L predicted contacts show 14%-23% lower precisions than that based on top L/5 predicted contacts, but folded structures based on top L predicted contacts show 0.06–0.227 higher TM-scores than that based on top L/5 predicted contacts. We then show tertiary structure prediction results in a more detailed manner in S12 Table. Although there are some exceptions (as shown by the underlined cases for α and β proteins in S12 Table), top L predicted contacts are overall more correlated with the quality of final structures than top L/2 and top L/5 predictions for different protein structural classes.

We also evaluate the impacts of all-range, mid-range, short-range and long-range contacts for 3D structure reconstruction. As shown in S10 and S11 Tables, long-range contacts contribute more to the quality of final protein models than short-range contacts. For the TestSet1_Tertiary1 test set, structure reconstructions achieve TM-scores of 0.31–0.58 and 0.23–0.237 for top L long- and short-range predictions, respectively. The results also show that the reconstructions based on top L all-range contacts achieve slightly better performance than that based on top L long-range contacts. The phenomenon indicates that all contacts may provide information about protein structure and important interactions, with shorter range contacts being useful for secondary and local structure, while longer range contacts are useful for determining global structure.

Finally, we show that higher precision in contact prediction cannot always lead to better performance in protein folding. The phenomenon can be investigated in the following three ways: (1) For the same contact prediction method evaluated over the same range and on the same test set, the prediction precisions for top L/5 contacts are higher than that for top L contacts, but the folding structures based on top L/5 predicted contacts show lower TM-scores that that based on top L predicted contacts, as indicated by the data from the last column in S10 and S11 Tables. (2) Comparing different contact prediction methods over the same range and on the same test set, ECA methods such as CCMpred can outperform DL methods for structure reconstruction based on top L/5 all- and long-range predicted contacts, even though DL methods show higher prediction precisions for residue contacts. This may be because that DL are more prone to predict contacts as patterns while the predictions from ECA methods are scattered along the sequence. So, when a limited number of all- and long-range predicted contacts are considered, ECA methods like CCMpred may generate more constraints of different topologies than DL methods. (3) We also investigate the correlation between TM-scores of folding structures and the precisions of corresponding contact predictions for different structural types of proteins in TestSet1_Tertiary1 (S12 Table). β and multi-domain proteins show higher contact prediction precisions than α proteins but lower folding structure TM-scores for top L, top L/2 and top L/5 predictions.

## Discussion

The sequence of a protein encodes the blueprint of its 3D structure. Accurate residue contact prediction from the sequence is critical for protein structure reconstruction. With the help of DCA and deep neural networks, accurate protein inter-residue contact prediction promotes the development of many leading protein structure prediction methods in recent years. Thus, a large-scale comprehensive assessment of contact prediction methods is very needed. Through a retrospective analysis on ML/ ECA methods and a multi-perspective study on recently developed DL methods, we try to answer four “what” questions regarding the contact prediction problem and work on some exploratory discussions on its application in 3D structure reconstruction.

What are the main factors that affect the performance of contact prediction? Various factors may affect the performance of contact prediction, and our assessment results demonstrate that the prediction accuracy depends on two main factors: contact density and the effective number of sequences in MSA. Contact density is the internal factor that impacts prediction accuracy because most methods make use of contact occurrence patterns[11,47]. When contact density is low, it is hard to identify reliable contact patterns. Based on the top L and L/5 predictions, correlation are visible for density values in the range of 0.0–2.0 and a strong correlation only lies in proteins with contact density 0.0–1.0. Among all the external factors, ***N***_***eff***_ of MSA shows the strongest impact on ECA and DL methods because it determines the quality of co-evolution signals. The quality of MSA can entirely decide the performance of ECA/ single-input DL methods and greatly affect the performance of consensus ML/multi-input DL methods. Most ECA and DL methods can obtain the best prediction results when *N*_*eff*_ > 5-8L.

What are the most overall advanced techniques for contact prediction? Benefiting from the ever-increasing sequence databases, diverse input features, well-designed networks and up-to-date large training sets, the extensively trained DL methods dominate current approaches in multiple different ways. The advantages of these DL methods lie in the overall prediction precisions, reliability of model probability, and robustness for low-contact-density and shallow *N*_*eff*_ proteins, etc. The complexity and high nonlinearity of the deep neural networks make the DL methods more expressive than ML methods. High expressivity requires more data to enhance the generalization ability of the network, so DL methods with large training sets and diverse features usually perform better than those with small training sets or limited feature categories. The overall prediction precision has been improved by around 45%/55% from traditional ML to multi-input DL methods for all-/long-range top L/5 predictions. Convincing results demonstrate that these DL methods show much higher reliability in model probability compared to ML and ECA methods. Further, the contact propensities derived from the model probability show potentials in predicting contacts in low-contact-density proteins and detecting the intrinsically disordered regions.

What are the application scenarios for different methods? Of all the methods evaluated here, no single tool is dominant in all the aspects of evaluation. In general, we make the following suggestions: (1) Multi-input DL methods are the best choices for protein types with low contact densities such as intrinsically disordered proteins and only a limited number of top predictions above a specific probability threshold are recommended to avoid the high risk of low precision. (2) Physicochemical interactions can be detected from predicted contacts. For top L/2 and L/5 predictions, DL methods can predict more hydrophobic interactions while ECA methods predict more salt bridges and disulfide bonds. (3) When evaluated on top L/*n* (*n* = 1, 2, 5) predictions, ECA methods can detect more secondary structure interactions, while DL methods can accurately excavate more contact patterns and prune isolated false positives. (4) With at least 5L-8L effective sequences in the multi-sequence alignment (MSA), all methods show the best performance and those methods that rely only on MSA as input can reach comparable achievements as methods that adopt multi-categories of inputs.

What are the bottlenecks and prospective directions for further development? Although DL methods show the highest overall prediction precisions, evidence still indicates much room left for improvement: (1) With a limited number of effective sequences in the MSA, the performance will be greatly affected. (2) Current methods show lower prediction precisions for inter-domain compared with intra-domain contact predictions, as well as very high imbalances in precisions for intra-domains. (3) Strong prediction similarities between DL methods indicating more feature types and diversified models need to be developed. (4) The typical time-consuming processes for current DL methods include the PSSM generation process, MSA-based covariance information extraction and integration of other contact prediction methods, and runtime optimization of most methods can be further improved.

The predicted residue contacts have many valuable applications in computational biology. In this study, we apply the predicted residue contacts from different methods to the tertiary structure reconstruction based on our test set with high sequence non-redundancy and structural diversity. We show that: (1) Although there are some exceptions for α and β proteins, top L predicted contacts are generally more correlated with the quality of final structures than top L/2 and top L/5 predictions for different protein structural classes. (2) All contacts from different sequence separations provide important interaction information for a protein’s overall structure, so folded structures based top L all-range contacts achieve overall better performance than that based on top L long-range contacts. Long-range contacts contribute more to the quality of final protein models than short-range and mid-range contacts. (3) When evaluated on top L/n (n = 1, 2, 5) contact predictions, higher precisions in contact prediction cannot always lead to better performance in contact-guided 3D structure reconstruction. A balance between contact prediction precision and region-distribution diversity should be considered, especially for multi-domain proteins and single-domain proteins rich in βsheets.

It should be noted that our analysis is based on the assumption that residue contacts are binaries, whereas distance contact prediction also shows promising practicality. Hence, similar assessments could be performed by considering contact distances.

## Supporting information

S1 Fig The overall prediction precision on TestSet2 for (a) short-range (b) medium-range and (c) long-range residue contacts. Superscripts *, **, ^◆^, ^†^ and ^††^ represent method categories of traditional-ML, consensus-ML, ECA, single-input DL and multi-input DL. DL methods significantly outperform ML and ECA methods for all contact ranges, and trRosetta/ RaptorX show close prediction precisions with SPOT.
(TIF)

S2 Fig Jaccard index and evolutionary relationship between 7 DL predictors. The Jaccard indices are calculated using top L predicted contacts for each protein in TestSet2 and then averaged on the whole TestSet2. The clustering results indicate that trRosetta and RaptorX show higher prediction similarity with SPOT than other DL methods.
(TIF)

S3 Fig Prediction similarity between different methods for individual proteins with Jaccard index > 0.5 on TestSet1. Each circle contains 16 arcs representing 16 different methods, and the corresponding arc of each method consists of 610 protein sites. Two sites of the same protein on different arcs will be linked together when the Jaccard index is greater than 0.5. The intended arc in each circle is the method used for analysis. Dense links can be observed between DL methods.
(TIF)

S4 Fig The plot of precisions for all-range predictions versus sequence length on TestSet1. Red dots and blue crosses indicate the targets for top L and top L/5 predictions, respectively. Pearson’s correlation coefficient (PCC) is used to measure the linear correlation between prediction precision and sequence length. Negative/ no/ positive/ positive correlation between precision and sequence can be observed for traditional ML/ consensus ML/ ECA/ DL methods, however, the correlations are not strong.
(TIF)

S5 Fig Prediction precisions of top L and L/5 contacts with the variation of contact density. From left to right the bar plots illustrate the prediction precisions for proteins (with ***N***_***eff***_ >L in TestSet1) with contact density >0.0/ 0.0–0.5/ 0.5–1.0/ 1.0–1.5/ 1.5–2.0/ 2.0–2.5. The dark-and light-colored bar in each sub-plot represent the precisions for top L and L/5 predictions, respectively. The error bar is the standard deviation of all precisions (for top L/5 predictions) in each sub-test set. Strong/weak positive correlations between precision and contact density can be found in Den 0.0–1.0 and Den 1.0–2.0.
(TIF)

S6 Fig Prediction coverages of 18 methods on TestSet2 for physicochemical interactions: (a) hydrophobic interactions (b) salt bridges (c) disulfide bridges. For top L/5 predictions, DL methods can predict more hydrophobic interactions while ECA methods predict more salt bridges and disulfide bonds.
(TIF)

S7 Fig Prediction proportions of true positive contacts for different secondary structure types on TestSet1. The internal pie and external ring and show the proportions of strand-strand, helix-helix, strand-helix, strand-loop, helix-loop, loop-loop in top L and L/5 true positive predicted contacts, respectively. As the number of predictions decreases, supervised techniques are more inclined to predict higher ratios of strand-strand contact types.
(TIF)

S8 Fig Secondary-structure-related prediction performance on TestSet2. (a) Prediction proportions of true positive contacts for different secondary structure types. The internal pie and external ring and show the proportions of strand-strand, helix-helix, strand-helix, strand-loop, helix-loop, loop-loop in top L and L/5 true positive predicted contacts, respectively. As the number of predictions decreases, supervised techniques are more inclined to predict higher ratio of strand-strand contact types. (b) The box plots from left to right show the prediction region (secondary structure interaction) coverage of top L, L/2 and L/5, respectively (the pink dashed line is the average prediction coverage of each method). DL methods make use of contact occurrence patterns for accurate prediction, thus the prediction is less dispersed on secondary-structure-based regions when a limited number of top predictions are considered.
(TIF)

S9 Fig Residue contact map of 2BZ1A by SVMSEQ/ gDCA/ trRosetta/ RaptorX for top L, L/2 and L/5 predictions. Gray squares in the left upper part are the contacts in native structures, blue squares in the right lower part is the correctly predicted contacts and red triangles are wrongly predicted contacts. Regions are divided by the black dashed lines centered with light green secondary structures. When a limited number of top predictions are considered, the predictions by DL methods cover fewer regions, but are more accurate in overall performance and contact pattern recognition.
(TIF)

S1 Table Summary of the evaluated methods. (XLSX)

S2 Table The prediction probability (score) ranges for 16 methods on TestSet1. (XLSX)

S3 Table Pearson’s correlation coefficient between prediction precision and contact density ranged from 0.0 to 2.5 (*N*_*eff*_ > L, TestSet1). (XLSX)

S4 Table Prediction precisions of top L, L/2 and L/5 all-range predictions by 16 methods on different protein types (*N*_*eff*_ > L, TestSet1). (XLSX)

S5 Table Prediction precision of DL methods on IDR proteins in TestSet1 through considering both top L/*n* and model probability strategies. (XLSX)

S6 Table Contact prediction precisions and salt bridge coverages of 5WK0A for top L/5 predictions. (XLSX)

S7 Table 108 multi-domain PDB chains in TestSet1 (26 underlined PDB chains are also in Testest2). (XLSX)

S8 Table Intra- and inter-domain prediction precisions of top L, L/2 and L/5 all-range predictions by 18 methods on TestSet2. (XLSX)

S9 Table Prediction probability of different methods for T0960-D1 and T0963-D1. (XLSX)

S10 Table Performance measured by TM-score of contact-guided 3D structure prediction on TestSet1_Tertiary1. Numbers inside the parentheses indicate the precisions of the corresponding predicted contacts.
(XLSX)

S11 Table Performance measured by TM-score of contact-guided 3D structure prediction on TestSet1_Tertiary2. Numbers inside the parentheses indicate the precisions of the corresponding predicted contacts.
(XLSX)

S12 Table Performance measured by TM-score of contact-guided 3D structure prediction for different protein structural types in TestSet1_Tertiary1. Numbers inside the parentheses indicate the precisions of the corresponding predicted contacts. The underlined numbers indicate cases that reconstructed structures based on top L/2 or top L/5 predicted contacts show higher TM-score than that based on top L predicted contacts.
(XLSX)
