# Supplementary figures and images for: Evaluation of residue-residue contact prediction methods: From retrospective to prospective
Source: PLoS Comput Biol. 2021 May 24;17(5):e1009027. doi: 10.1371/journal.pcbi.1009027 (PMC8177648; doi:10.1371/journal.pcbi.1009027)

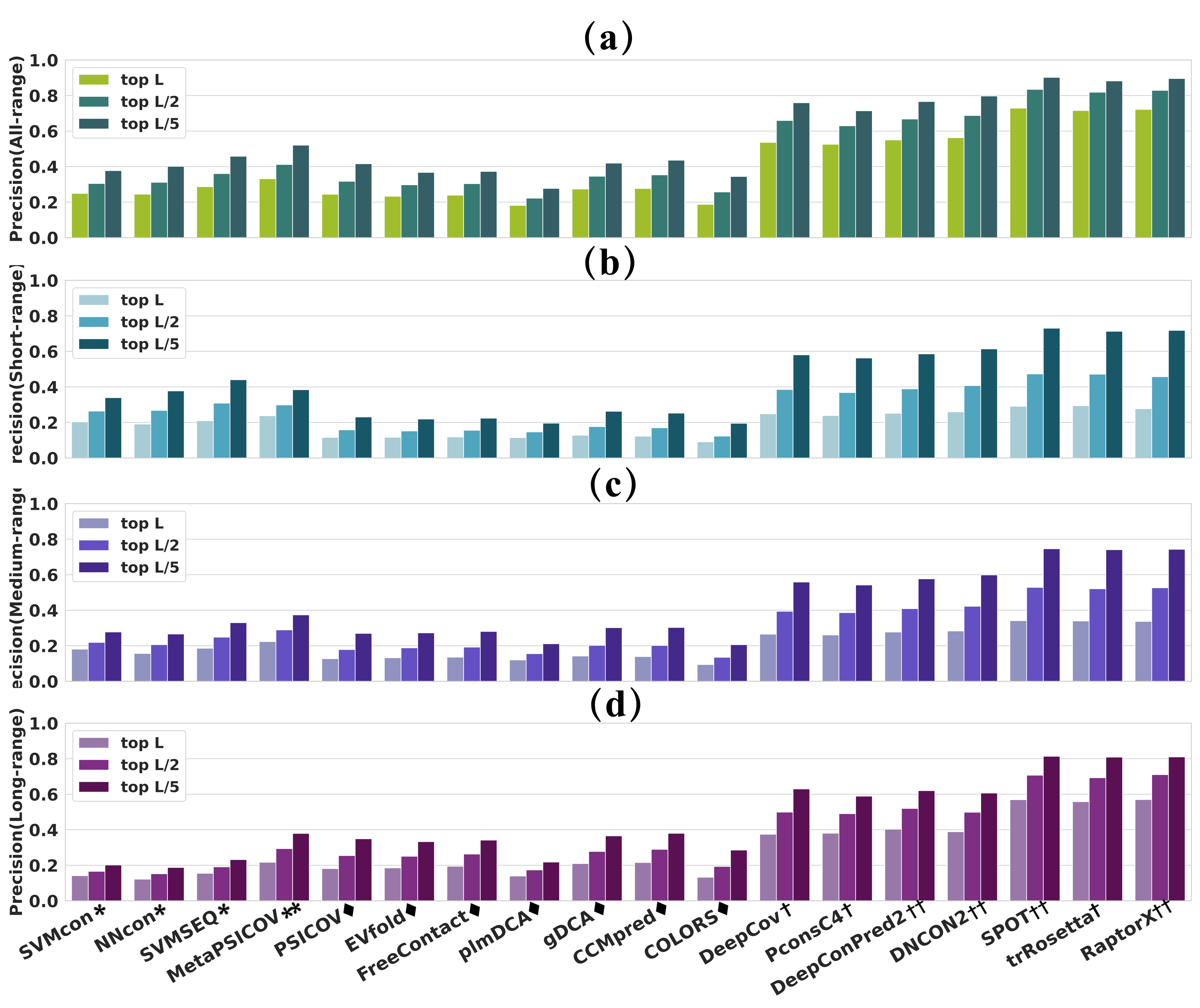

Supplement: S1 Fig — The overall prediction precision on TestSet2 for (a) short-range (b) medium-range and (c) long-range residue contacts. Superscripts *, **, ◆, † and †† represent method categories of traditional-ML, consensus-ML, ECA, single-input DL and multi-input DL. DL methods significantly outperform ML and ECA methods for all contact ranges, and trRosetta/ RaptorX show close prediction precisions with SPOT. (TIF) [file pcbi.1009027.s001.tif]

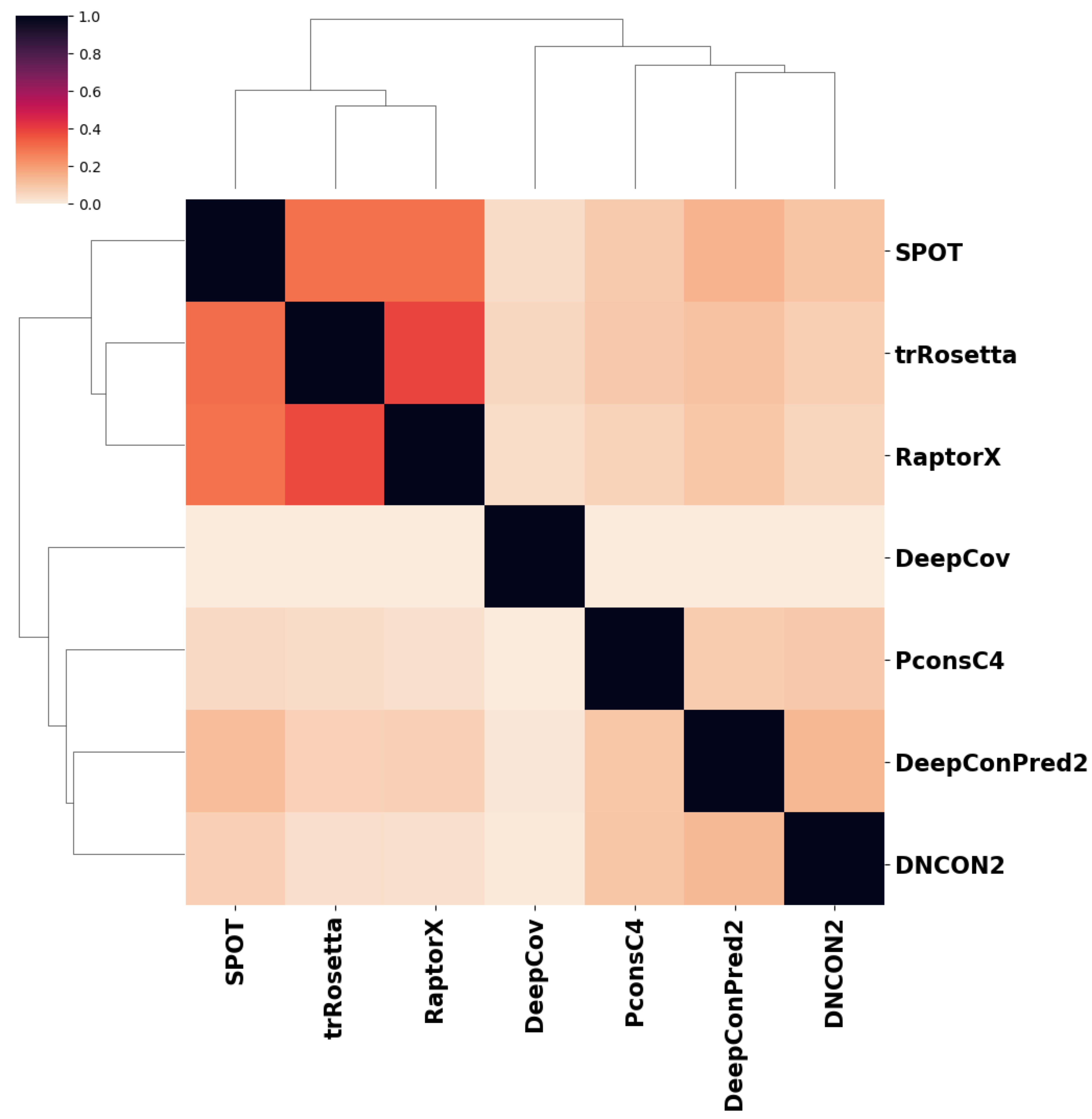

Supplement: S2 Fig — The Jaccard indices are calculated using top L predicted contacts for each protein in TestSet2 and then averaged on the whole TestSet2. The clustering results indicate that trRosetta and RaptorX show higher prediction similarity with SPOT than other DL methods. (TIF) [file pcbi.1009027.s002.tif]

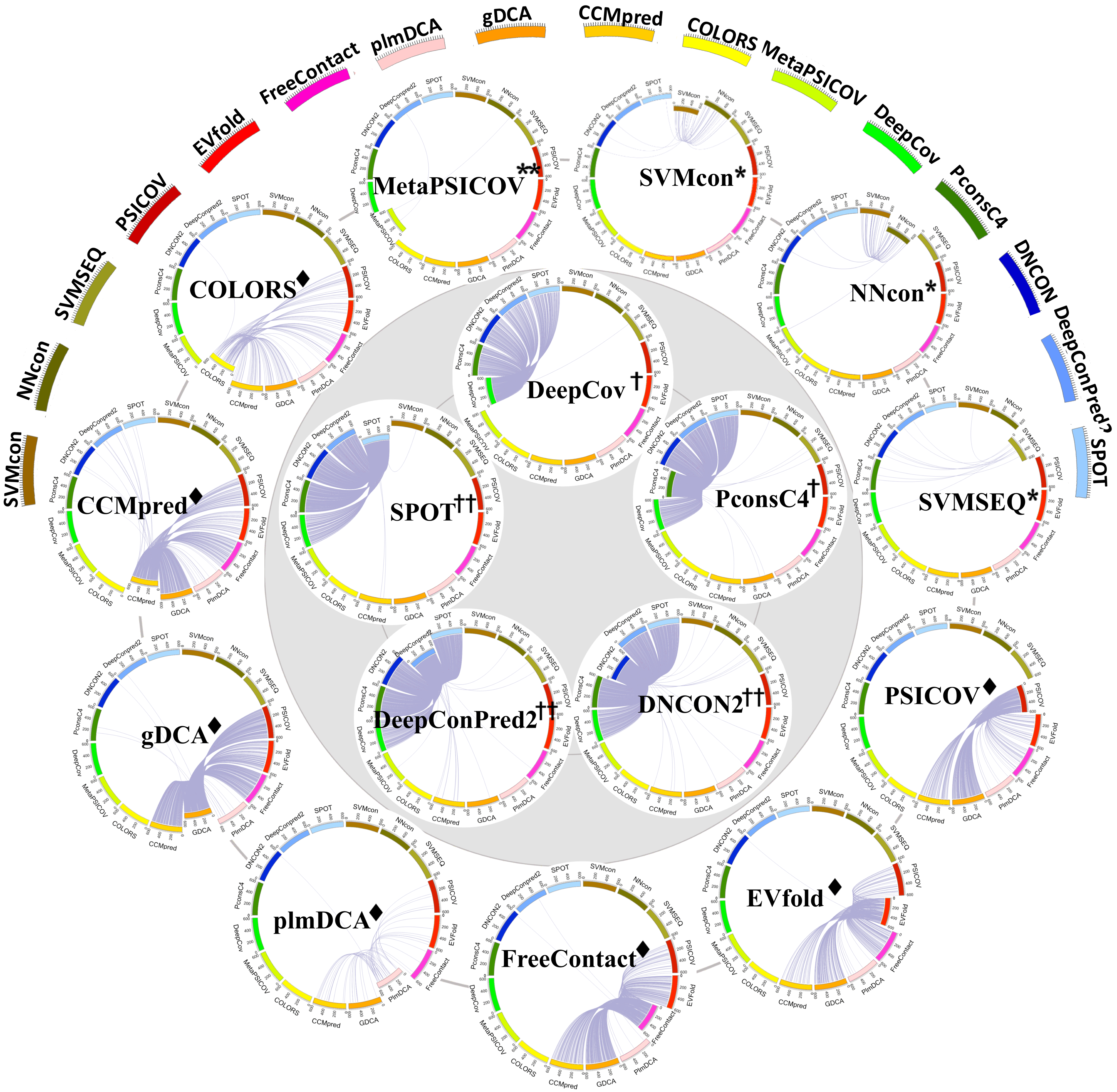

Supplement: S3 Fig — Each circle contains 16 arcs representing 16 different methods, and the corresponding arc of each method consists of 610 protein sites. Two sites of the same protein on different arcs will be linked together when the Jaccard index is greater than 0.5. The intended arc in each circle is the method used for analysis. Dense links can be observed between DL methods. (TIF) [file pcbi.1009027.s003.tif]

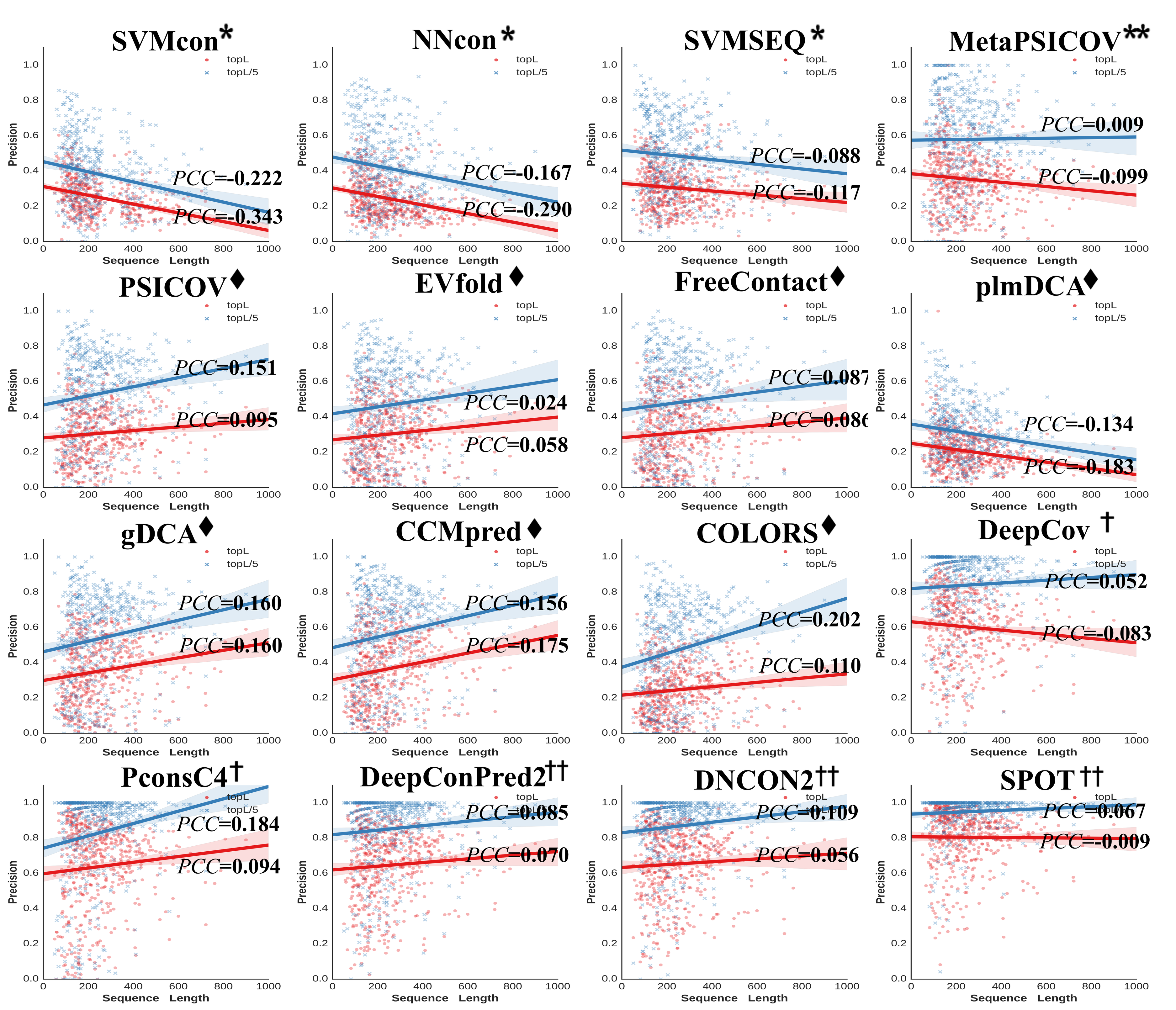

Supplement: S4 Fig — Red dots and blue crosses indicate the targets for top L and top L/5 predictions, respectively. Pearson’s correlation coefficient (PCC) is used to measure the linear correlation between prediction precision and sequence length. Negative/ no/ positive/ positive correlation between precision and sequence can be observed for traditional ML/ consensus ML/ ECA/ DL methods, however, the correlations are not strong. (TIF) [file pcbi.1009027.s004.tif]

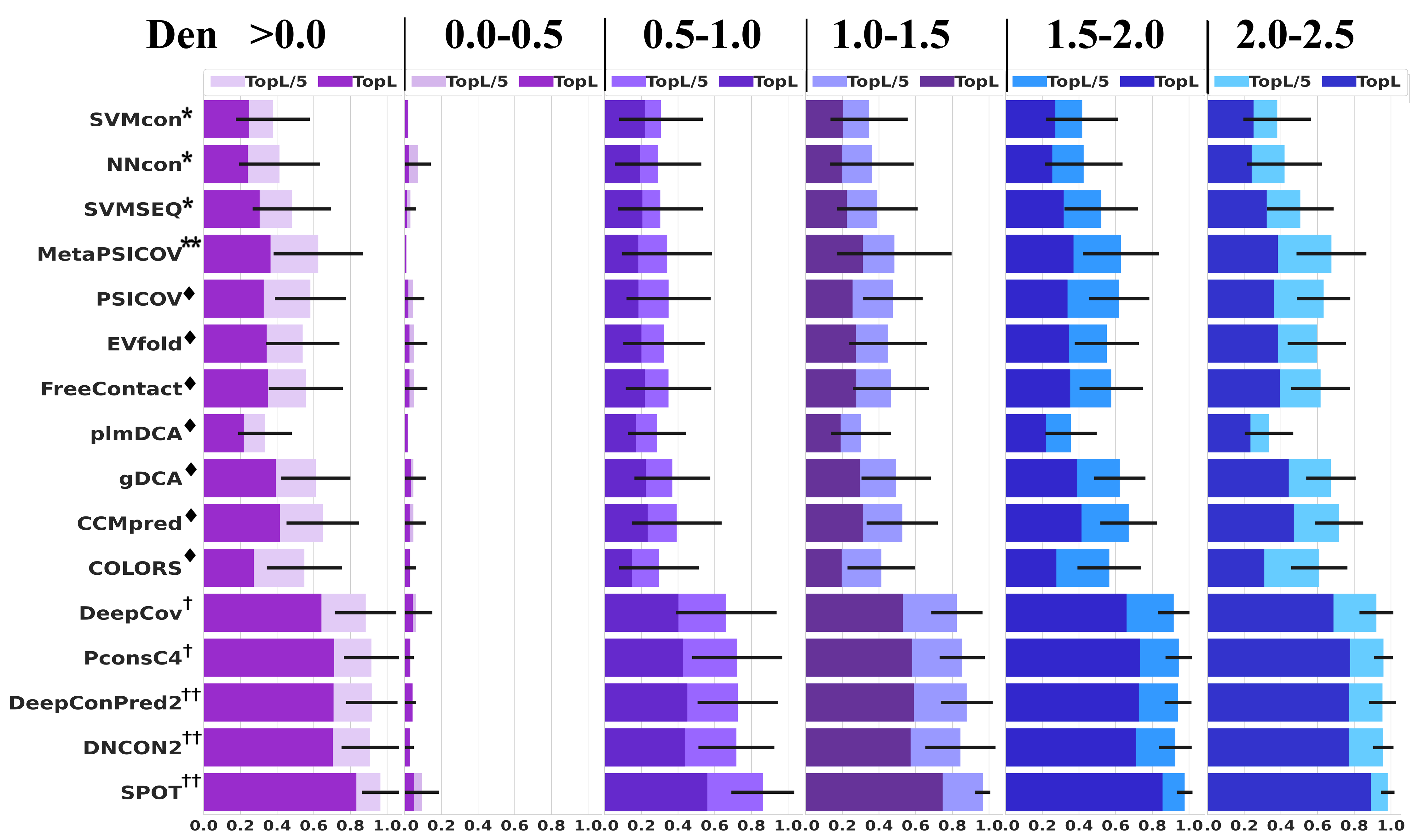

Supplement: S5 Fig — From left to right the bar plots illustrate the prediction precisions for proteins (with Neff >L in TestSet1) with contact density >0.0/ 0.0–0.5/ 0.5–1.0/ 1.0–1.5/ 1.5–2.0/ 2.0–2.5. The dark-and light-colored bar in each sub-plot represent the precisions for top L and L/5 predictions, respectively. The error bar is the standard deviation of all precisions (for top L/5 predictions) in each sub-test set. Strong/weak positive correlations between precision and contact density can be found in Den 0.0–1.0 and Den 1.0–2.0. (TIF) [file pcbi.1009027.s005.tif]

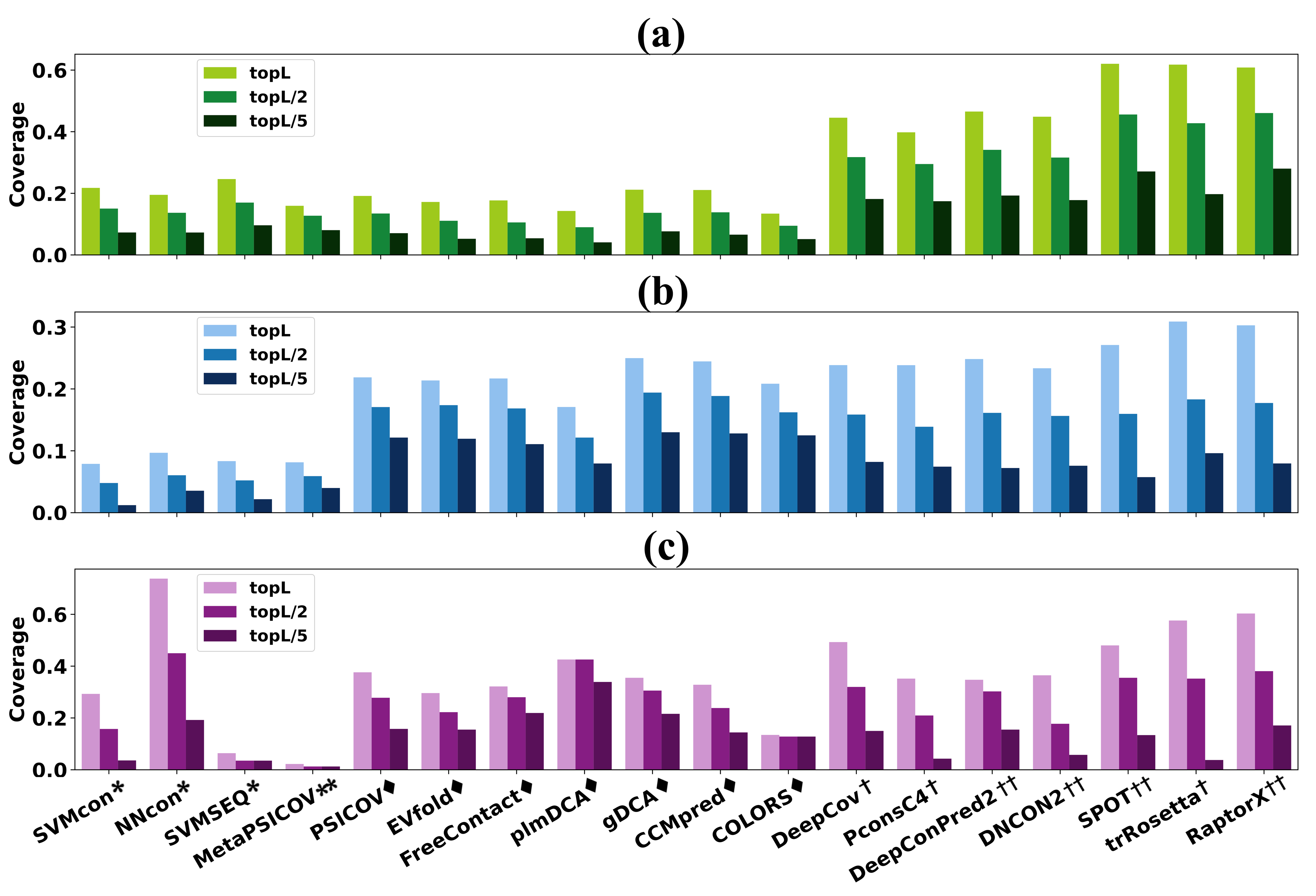

Supplement: S6 Fig — Prediction coverages of 18 methods on TestSet2 for physicochemical interactions: (a) hydrophobic interactions (b) salt bridges (c) disulfide bridges. For top L/5 predictions, DL methods can predict more hydrophobic interactions while ECA methods predict more salt bridges and disulfide bonds. (TIF) [file pcbi.1009027.s006.tif]

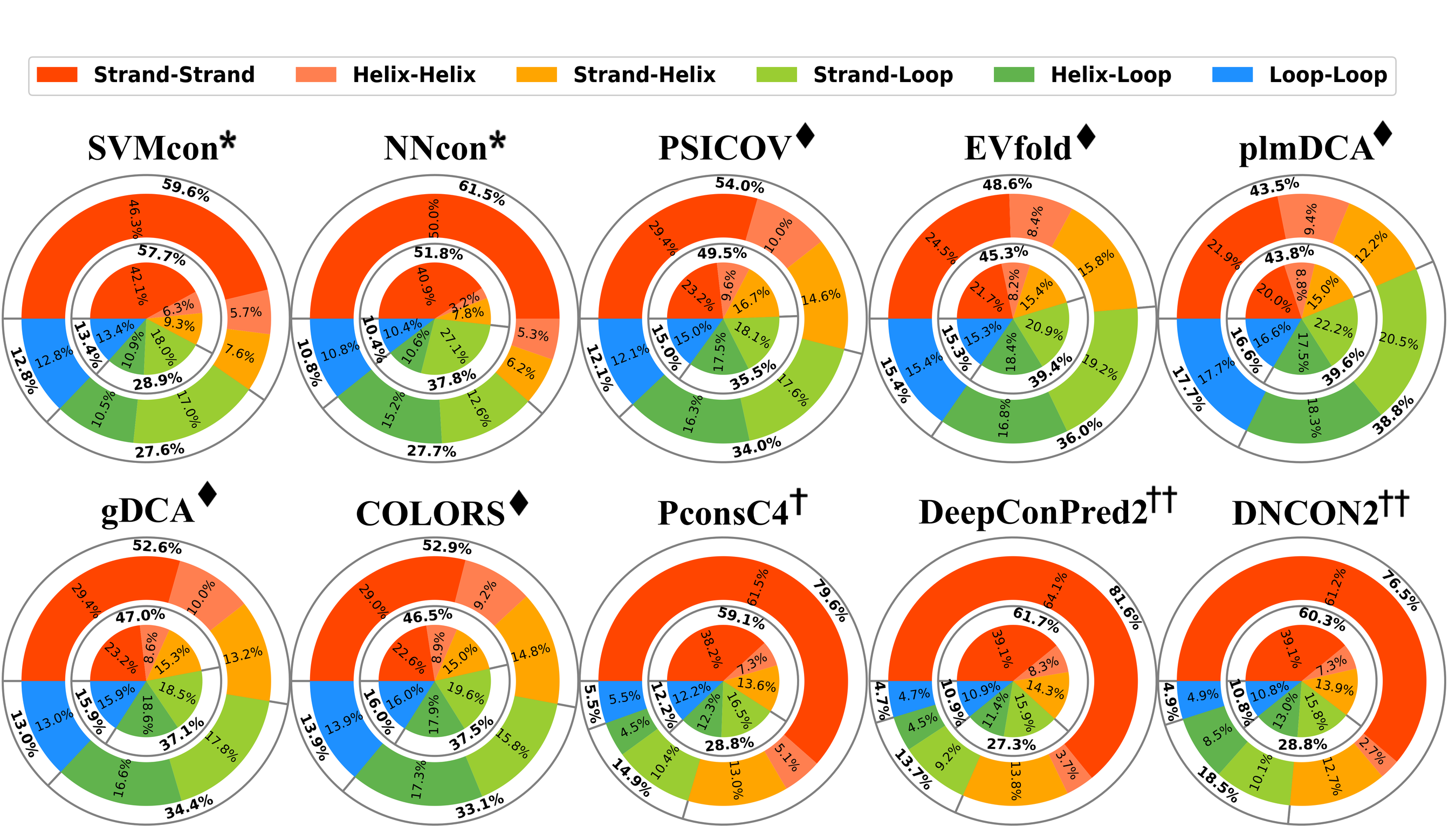

Supplement: S7 Fig — The internal pie and external ring and show the proportions of strand-strand, helix-helix, strand-helix, strand-loop, helix-loop, loop-loop in top L and L/5 true positive predicted contacts, respectively. As the number of predictions decreases, supervised techniques are more inclined to predict higher ratios of strand-strand contact types. (TIF) [file pcbi.1009027.s007.tif]

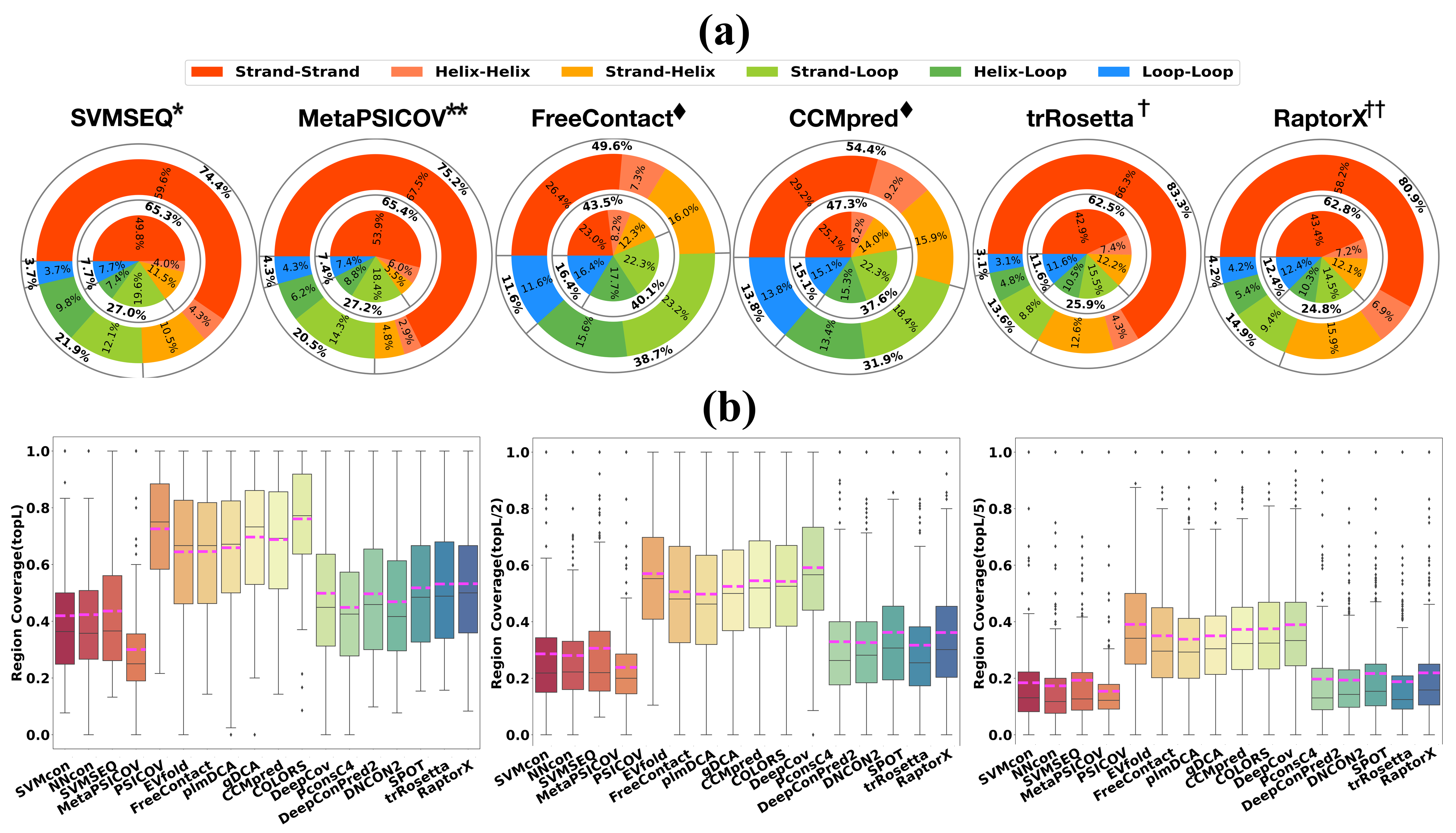

Supplement: S8 Fig — (a) Prediction proportions of true positive contacts for different secondary structure types. The internal pie and external ring and show the proportions of strand-strand, helix-helix, strand-helix, strand-loop, helix-loop, loop-loop in top L and L/5 true positive predicted contacts, respectively. As the number of predictions decreases, supervised techniques are more inclined to predict higher ratio of strand-strand contact types. (b) The box plots from left to right show the prediction region (secondary structure interaction) coverage of top L, L/2 and L/5, respectively (the pink dashed line is the average prediction coverage of each method). DL methods make use of contact occurrence patterns for accurate prediction, thus the prediction is less dispersed on secondary-structure-based regions when a limited number of top predictions are considered. (TIF) [file pcbi.1009027.s008.tif]

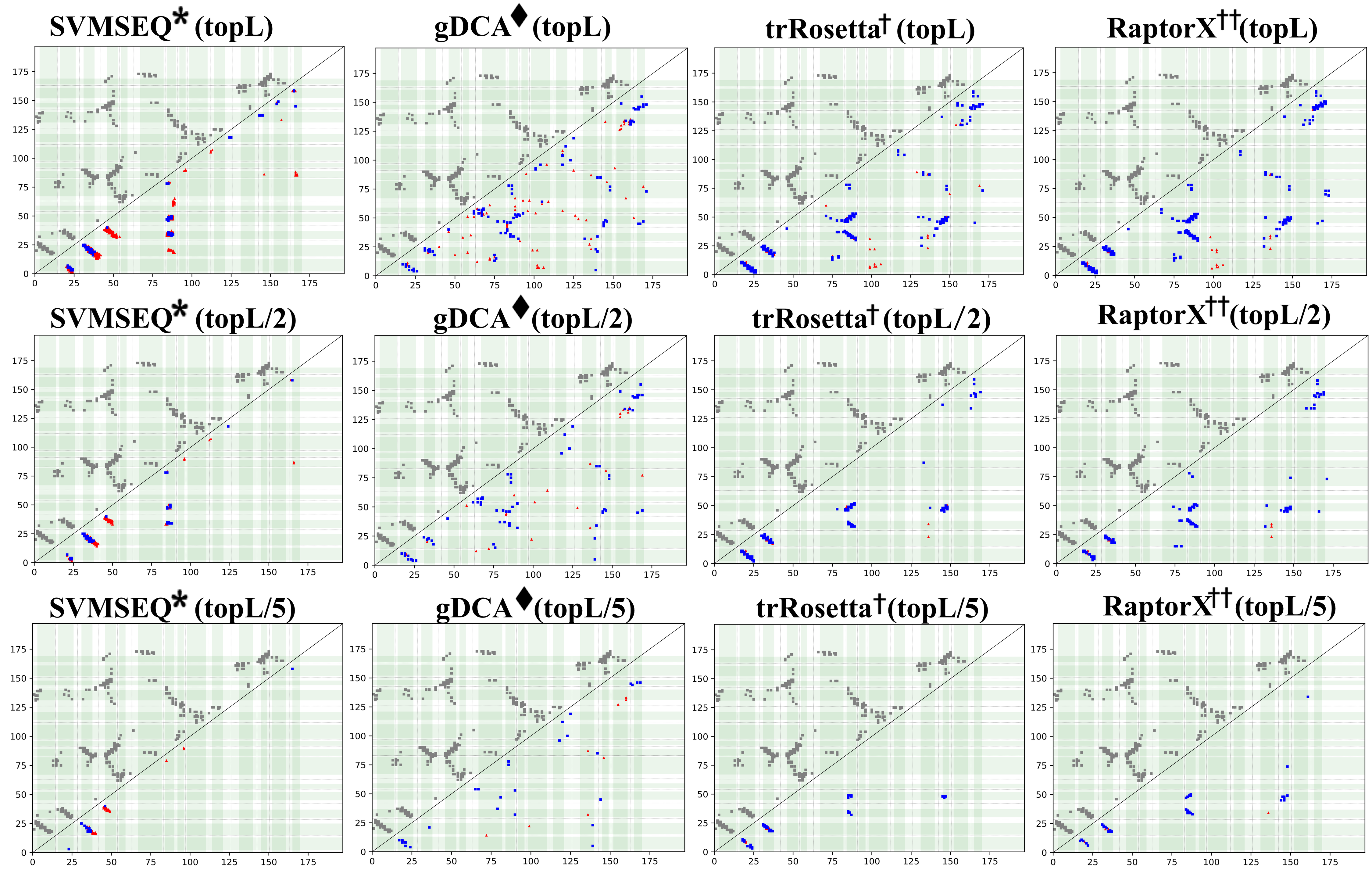

Supplement: S9 Fig — Gray squares in the left upper part are the contacts in native structures, blue squares in the right lower part is the correctly predicted contacts and red triangles are wrongly predicted contacts. Regions are divided by the black dashed lines centered with light green secondary structures. When a limited number of top predictions are considered, the predictions by DL methods cover fewer regions, but are more accurate in overall performance and contact pattern recognition. (TIF) [file pcbi.1009027.s009.tif]
